# Recent advances in biomarkers for cardiac fibrosis

**DOI:** 10.3389/fcvm.2026.1874539

**Published:** 2026-09-08

**Authors:** Xiduo Zhao, Lianyu Hua, Youjun Xiong, Fuyou Shi, Yilin Zhong, Shangwei Zhang, Chengnan Tian

**Affiliations:** 1 The First Clinical College, Gannan Medical University, Ganzhou, Jiangxi, China; 2 Department of Cardiovascular Surgery, The First Affiliated Hospital of Gannan Medical University, Ganzhou, Jiangxi, China

**Keywords:** biomarkers, cardiac fibrosis, cardiac magnetic resonance imaging, extracellular matrix, prognosis prediction

## Abstract

Myocardial fibrosis significantly contributes to the progression of cardiovascular diseases, ultimately leading to heart failure. Early, non-invasive diagnosis is essential for improving patient outcomes. This article reviews recent qualitative research on myocardial fibrosis biomarkers from 2022 to 2026, focusing on descriptive integration of clinical observational evidence without conducting a unified quantitative comparison of diagnostic efficacy. Initially, the article outlines the pathophysiological background of myocardial fibrosis, emphasizing both canonical TGF-*β*/Smad and non-canonical signaling pathways (MAPK, PI3K/Akt, Rho-like GTPase) that drive fibrotic remodeling. It further analyzes the interactions and feedback loops among core fibrogenic signaling pathways, providing a basis for interpreting dynamic changes in biomarker levels over time. The review examines current research progress, potential clinical applications, and limitations associated with circulating protein biomarkers, cardiac magnetic resonance imaging biomarkers, and emerging multi-omics molecular biomarkers. It distinguishes between biomarkers that indicate irreversible scar tissue and those that detect active, reversible fibrotic processes, clarifying their suitability for early disease detection and monitoring established disease. Furthermore, the article addresses the mechanisms of exosomal miRNA and protein packaging in activated cardiac fibroblasts and their release into peripheral circulation, elucidating the cellular origin and function of these circulating biomarkers. It also discusses the impact of post-translational modifications, such as glycosylation and phosphorylation, on biomarker stability, assay performance, and functional activity, offering insights for assay optimization and result interpretation. The review critically examines the correlation between circulating biomarkers and invasive endomyocardial biopsy-based histopathological fibrosis quantification, the diagnostic gold standard, and analyzes sources of heterogeneity in existing validation studies. It elaborates on molecular mechanisms, detection technologies, clinical validation results, and the practical value of these biomarkers in patient risk stratification, treatment response monitoring, and prognosis prediction. Specifically, it summarizes changes in circulating and imaging biomarkers following interventions like renin-angiotensin-aldosterone system inhibitors, angiotensin receptor-neprilysin inhibitors, and sodium-glucose cotransporter 2 inhibitors. The article analyzes evidence on the temporal relationship between biomarker trajectories and cardiac functional improvements. Finally, the article explores strategies for multi-biomarker combined evaluation, proposing a framework for panel design that considers biomarker weighting, cross-class combination, redundancy reduction, and cost-performance balance. It discusses future directions for clinical translational research, aiming to provide a theoretical reference and novel perspectives for the comprehensive assessment and precise management of myocardial fibrosis.

## 1 Introduction

Myocardial fibrosis is the heart's adaptive response to chronic stressors like pressure or volume overload, ischemia, and inflammation (1, 2). This condition is characterized by the excessive activation and proliferation of cardiac fibroblasts, which secrete substantial amounts of extracellular matrix proteins (3). This secretion leads to myocardial tissue stiffening, disrupts electrical conduction, and impairs both systolic and diastolic functions (4). Myocardial fibrosis plays a crucial role in diseases such as hypertensive heart disease, hypertrophic cardiomyopathy, myocardial infarction, and the progression of heart failure (5). While endomyocardial biopsy is the diagnostic gold standard, its invasive nature limits its routine clinical use (6). Most non-invasive biomarker validation studies have relied on cardiac magnetic resonance parameters as a reference standard instead of direct histological quantification. Consequently, identifying reliable, non-invasive biomarkers with strong histological correlation has become a major focus in cardiovascular research (7). Notably, the concordance between circulating fibrosis markers and biopsy-derived collagen volume fraction varies significantly across published cohorts, and the methodological factors contributing to this inconsistency have not been systematically reviewed. Earlier reviews mainly discuss classic biomarkers identified before 2022, such as collagen metabolites PICP/PIII-NP, Gal-3, thrombospondin-2, and traditional CMR indicators (8). These reviews often describe findings without differentiating between incremental progress and original innovations. Since 2022, advancements in high-throughput sequencing, artificial intelligence (AI) imaging analysis, and multi-center prospective cohort studies have led to significant breakthroughs (9). New regulatory molecules in fibrosis signaling pathways, optimized imaging biomarker detection protocols, tissue-specific non-coding RNA panels, and multi-omics combined models have moved from basic research to clinical validation.

The mechanisms underlying myocardial fibrosis are intricate, involving a variety of cytokines, signaling pathways, and cell types (10, 11). Central to this pathological process is the TGF-*β* signaling cascade, with downstream Smad proteins playing a crucial role in fibroblast activation and extracellular matrix production (5). Research indicates that the TGF-*β*1/Smad pathway is activated in diabetic cardiomyopathy models, and its inhibition can reduce myocardial fibrosis (12). Beyond the classical TGF-*β* pathway, molecules such as thrombospondin-2 are gaining attention (13, 14). Thrombospondin-2, part of the interleukin-1 receptor family, is closely linked to myocardial fibrosis and is considered a potential biomarker for its assessment (15). In patients with cardiovascular disease, serum thrombospondin-2 levels positively correlate with the severity of myocardial fibrosis, offering diagnostic value (16). Recent studies have identified multiple epigenetic regulators and metabolic enzymes that mediate pathway activity in a tissue-specific manner (17). This discovery helps explain the inconsistent correlation of classic circulating markers across different patient cohorts in recent clinical trials. Additionally, non-coding RNAs, particularly microRNAs (miRNAs) and circular RNAs (circRNAs), have shown significant breakthroughs in clinical sample validation since 2022. Unlike early basic research, recent studies confirm that circRNAs and miRNAs can stably regulate myocardial fibrosis-related genes at both transcriptional and post-transcriptional levels in peripheral blood. These findings suggest they have higher diagnostic specificity than traditional collagen metabolites, making them a promising focus for early diagnosis research (18). Notably, most early cohort analyses failed to separate the independent prognostic effect of these novel molecules from conventional clinical confounders. However, subsequent post-2022 multivariate adjusted models have clarified that non-coding RNAs still serve as independent predictors of adverse cardiovascular events after full correction for classic risk factors, rather than simply mirroring disease severity captured by routine clinical indicators.

In clinical practice, the evaluation of myocardial fibrosis is transitioning from invasive biopsies to non-invasive, multimodal methods. Serum biomarkers are particularly advantageous due to their ease of collection and reproducibility. Collagen metabolites, such as pro-collagen type I carboxy-terminal peptide (PICP) and pro-collagen type III amino-terminal peptide (PIII-NP), serve as indirect indicators of type I and III collagen synthesis. These metabolites correlate closely with the extent of myocardial fibrosis in heart failure patients. However, multi-center studies conducted after 2022 have yielded mixed results. While some cohorts confirmed the predictive efficacy of these biomarkers, large-sample retrospective studies in specific populations, such as the elderly and those with liver or renal insufficiency, reported a notable decline in diagnostic effectiveness. The primary limitation identified was organ non-specificity, which hampers clinical application. In imaging, cardiac magnetic resonance imaging (CMR), especially T1 mapping and extracellular volume fraction (ECV), has seen significant advancements since 2022 (19). Innovations include optimized rapid scanning sequences and AI-assisted automatic quantification algorithms, which substantially reduce detection time and inter-observer variability. Comparative studies reveal that combining serum ferritin with ECV detection achieves an AUC of 0.89 for early diagnosis of post-myocardial infarction fibrosis. This combination outperforms single-indicator methods by providing mechanistically complementary diagnostic information rather than merely statistical enhancement. Emerging omics technologies, such as proteomics and metabolomics, have advanced beyond preliminary screening (20). Researchers have developed intermediate phenotypic molecule (IMP) risk scoring models targeting anthracycline cardiotoxicity and hypertensive myocardial fibrosis. These models have been externally validated in independent cohorts, marking a shift from omics screening to practical clinical risk assessment tools (21). All IMP models were constructed using multivariate Cox regression adjusted for core clinical risk factors. The consistent elevation of the C-index after biomarker inclusion confirms that multi-omics signatures provide unique prognostic information, supplementing standard clinical prediction models.

## 2 Literature search strategy, inclusion criteria and time range

This manuscript presents a narrative review emphasizing the qualitative integration and mechanistic interpretation of myocardial fibrosis biomarkers, distinct from a PRISMA-compliant systematic review that includes quantitative synthesis. To facilitate a comprehensive thematic discussion, we conducted targeted literature searches across four major biomedical databases: PubMed, Web of Science, Embase, and Scopus. Our primary focus is on clinical validation studies of novel fibrosis biomarkers within the core retrieval window of January 2022 to January 2026. Landmark mechanistic papers published before 2022 were selectively included to provide background on key fibrotic signaling pathways and classical biomarker standards. The retrieval strategy employed a combination of Medical Subject Headings (MeSH) and free-text keywords, including cardiac fibrosis, biomarkers, extracellular matrix, cardiac magnetic resonance imaging, and prognosis prediction. Literature screening adhered to flexible qualitative criteria aligned with the thematic focus of this narrative review. We prioritized manuscripts for citation based on the following criteria: (1) peer-reviewed original research, clinical trials, or high-quality thematic reviews centered on myocardial fibrosis biomarkers; (2) full-text English articles with complete experimental designs and clear, interpretable research outcomes; and (3) significant foundational mechanistic studies published before 2022 that are essential for elucidating core fibrogenic pathways. Publications were excluded from the narrative synthesis if they: (1) focused on fibrosis in non-cardiac organs; (2) were repetitive, low-quality reviews lacking novel mechanistic or clinical insights; or (3) consisted of conference abstracts, standalone case reports, editorial letters, or studies with incomplete raw data that could not support an in-depth discussion of mechanisms.

## 3 Pathophysiological basis and biomarker classification of myocardial fibrosis

### 3.1 Key signaling pathways and targets in fibrosis activation

**Figure 1:**
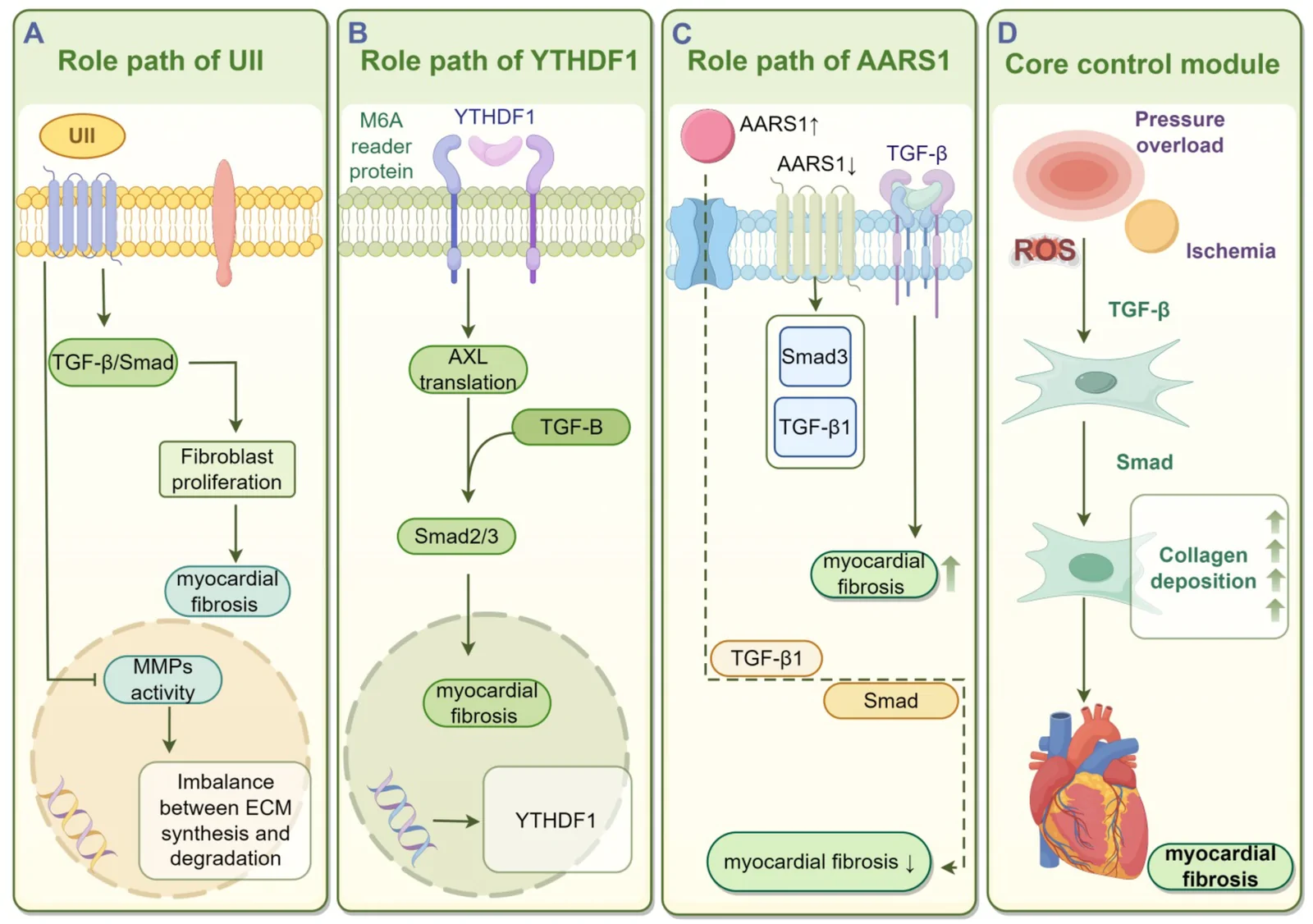
**(A)** during stress-induced cardiac hypertrophy, elevated levels of urocortin II (UII) stimulate cardiac myofibroblast proliferation by activating the TGF-*β*/smad signaling pathway. Simultaneously, UII inhibits matrix metalloproteinase (MMP) activity, disrupting the balance between extracellular matrix production and degradation, which ultimately promotes fibrosis. **(B)** The m6A reader protein YTHDF1 enhances AXL translation, subsequently activating the TGF-*β*–Smad2/3 pathway. This activation accelerates both the onset and progression of cardiac fibrosis. **(C)** A deficiency in aminoacyl-tRNA synthetase-1 (AARS1) activates the TGF-*β*1/Smad3 axis, promoting cardiac fibrosis. In contrast, increased AARS1 expression can inhibit this signaling pathway, thereby reducing fibrotic responses. **(D)** In various cardiovascular disorders, pathological factors such as mechanical stress, ischemia, and inflammation can activate the TGF-*β*/Smad pathway. This activation leads to collagen accumulation and extracellular matrix remodeling, fostering the development of myocardial fibrosis.

The transforming growth factor-*β* (TGF-*β*)/Smad pathway is a key profibrotic signaling axis in myocardial fibrosis, playing a crucial role in collagen deposition and extracellular matrix remodeling in various cardiovascular diseases. Studies indicate that urocortin II (UII) is upregulated in pressure-induced cardiac hypertrophy, where it promotes myocardial fibroblast proliferation and fibrotic responses by activating the TGF-*β*/Smad signaling pathway (22). This activation leads to increased collagen gene expression and the inhibition of matrix metalloproteinases (MMPs), thereby contributing to fibrosis. RNA epigenomic studies have further clarified the regulatory network of the TGF-*β*/Smad pathway. For instance, the m6A reader protein YTHDF1 enhances AXL translation, which in turn activates the TGF-*β*-Smad2/3 signaling pathway, worsening myocardial fibrosis (23). Additionally, the absence of aminoacyl-tRNA synthetase-1 (AARS1) is associated with increased myocardial fibrosis through the activation of the TGF-*β*1/Smad3 pathway. Conversely, upregulating AARS1 inhibits this pathway and reduces fibrosis (24). These findings underscore the central role of the TGF-*β*/Smad pathway and suggest that upstream regulators like UII and YTHDF1, along with the downstream effector molecule AARS1, could serve as therapeutic targets (Figure 1).

Beyond the canonical Smad-dependent cascade, TGF-*β* also promotes myocardial fibrosis through three well-defined non-canonical signaling pathways: mitogen-activated protein kinase (MAPK), phosphoinositide 3-kinase/protein kinase B (PI3K/Akt), and Rho-like GTPase pathways (25). These pathways function independently of Smad transcription factors and mediate distinct fibroblast activation phenotypes, providing unique molecular targets for developing pathway-specific biomarkers that capture fibroblast activation beyond canonical signaling (26). The MAPK signaling cascade, comprising p38 MAPK, extracellular signal-regulated kinase 1/2 (ERK1/2), and c-Jun N-terminal kinase (JNK), is rapidly activated by TGF-*β* through TGF-*β*-activated kinase 1 (TAK1) (27). Clinical cohort studies post-2022 have shown that phosphorylated p38 MAPK levels in peripheral blood mononuclear cells correlate with diffuse myocardial fibrosis in hypertensive heart disease and diabetic cardiomyopathy, with a diagnostic AUC comparable to Gal-3. Unlike systemic collagen metabolites, phosphorylated MAPK subtypes directly reflect intracellular fibroblast activation, enabling earlier detection of profibrotic signaling before extracellular matrix accumulation becomes measurable (28). Moreover, ERK1/2 pathway activity is closely linked to fibroblast proliferation and transition to myofibroblasts, making its downstream effectors promising for monitoring active fibrotic progression (29). The PI3K/Akt signaling axis is another crucial non-canonical branch mediating TGF-*β*-induced fibroblast survival, metabolic reprogramming, and extracellular matrix production. Recent proteomic studies have found elevated levels of circulating phosphorylated Akt (p-Akt) and its downstream effector, mammalian target of rapamycin (mTOR), in patients with progressive myocardial fibrosis, correlating with extracellular volume fraction (ECV) values measured by CMR (30). Notably, the PI3K/Akt pathway is predominantly activated in metabolic cardiomyopathy-related fibrosis, such as in diabetic and obese populations, offering superior etiological specificity for distinguishing metabolic-driven fibrosis from ischemic or hypertensive phenotypes. Rho-like GTPases, mainly RhoA, Rac1, and Cdc42, transmit TGF-*β* signals to regulate cytoskeletal remodeling, fibroblast migration, and contractile phenotype acquisition. The RhoA/Rho-associated coiled-coil containing protein kinase (ROCK) axis, in particular, has garnered significant attention as a therapeutic and biomarker target (31). Circulating RhoA activity and downstream ROCK1 expression levels strongly correlate with left ventricular diastolic dysfunction and interstitial fibrosis severity in heart failure with preserved ejection fraction (HFpEF). Since Rho/ROCK signaling primarily mediates mechanical stress-induced fibroblast activation, its associated biomarkers can specifically capture pressure overload-driven fibrotic remodeling, complementing the canonical Smad pathway markers that broadly reflect collagen synthesis.

Recent single-molecule studies reveal a complex mechanistic framework where UII, YTHDF1, and AARS1 regulate the TGF-*β*/Smad pathway at different levels, rather than forming a direct protein complex. These molecules orchestrate the fibrogenic cascade through three regulatory tiers: 1. Extracellular Initiation Tier: Urotensin II (UII), a secreted vasoactive peptide, initiates the signaling cascade by binding to its G-protein-coupled receptor, UT, on cardiac fibroblasts (32). This interaction upregulates TGF-*β*1 expression and activates latent TGF-*β* in the extracellular matrix, triggering profibrotic signaling at the tissue level. UII also indirectly increases YTHDF1 expression in fibroblasts via the ERK pathway, linking extracellular signals to intracellular epigenetic regulation. 2. Epitranscriptomic Amplification Tier: The m⁶A reader protein YTHDF1 amplifies signals at the post-transcriptional/translational level (33). It recognizes m⁶A-modified mRNA transcripts of key TGF-*β* pathway components, such as AXL and TGF-*β* receptors, enhancing their translation efficiency and boosting cellular responsiveness to TGF-*β* (34). YTHDF1 does not directly activate the pathway but reduces the activation threshold for downstream signaling in response to UII-induced TGF-*β*. Additionally, YTHDF1 modulates AARS1 mRNA translation in an m⁶A-dependent manner, linking the epitranscriptomic and metabolic effector layers (35). 3. Intracellular Metabolic Effector Tier: Aminoacyl-tRNA synthetase-1 (AARS1) serves as a downstream metabolic effector, directly influencing the activity of the Smad3 transcription factor. Loss of AARS1 enhances Smad3 nuclear translocation and transcriptional activity, increasing collagen synthesis and fibroblast activation initiated by UII and TGF-*β* signals. Conversely, increased AARS1 levels restrain excessive Smad3-mediated fibrotic responses, acting as a negative feedback mechanism (36). As a metabolic enzyme involved in protein synthesis, AARS1 links fibrotic signaling to the cellular metabolic state necessary for robust extracellular matrix production.

Since 2022, accumulating evidence has highlighted the functional roles and clinical biomarker potential of non-canonical TGF-*β* branches, shifting research from single pathway studies to an integrated analysis of the entire TGF-*β* signaling network (37). This research primarily focuses on understanding upstream regulatory factors and the interaction between canonical and non-canonical pathways. Beyond the TGF-*β* signaling cascade, three additional core profibrotic pathways—Wnt/*β*-catenin, Notch, and Hippo/YAP—function as independent yet equally significant drivers of myocardial fibrosis. These pathways offer distinct targets for pathway-specific biomarker development, aiming for comprehensive pathway coverage. The Wnt/*β*-catenin signaling axis is crucial in myocardial fibrosis induced by pressure overload and ischemia. Upon activation, *β*-catenin moves into the nucleus, upregulating profibrotic genes and promoting cardiac fibroblast proliferation and myofibroblast differentiation. This pathway closely interacts with the TGF-*β* cascade through Smad/*β*-catenin complex formation, amplifying fibrotic responses synergistically (38). Secreted frizzled-related protein 1 (sFRP-1) and Dickkopf-1 (Dkk-1) serve as endogenous Wnt antagonists, with their serum levels inversely correlating with myocardial fibrosis severity. Clinical studies post-2022 confirm that changes in circulating sFRP-1 levels accurately reflect the anti-fibrotic efficacy of sacubitril/valsartan, making it a promising surrogate endpoint for monitoring therapy response (39). The Notch signaling pathway, mediated by interactions between Jagged/Delta-like ligands and Notch receptors (Notch1–4), is another key regulator of fibroblast phenotypic transition. Activation of Notch1 and Notch3 in cardiac fibroblasts directly induces α-smooth muscle actin expression and extracellular matrix production, independent of TGF-*β* signaling. Recent cohort studies validate circulating soluble Jagged1 and the intracellular domain of Notch1 (N1ICD) as candidate biomarkers (40). Their levels are significantly elevated in patients with hypertensive and diabetic myocardial fibrosis, providing additional diagnostic value when combined with classic collagen turnover markers, especially for identifying metabolic-dominant fibrotic phenotypes. The Hippo/YAP signaling pathway is the core mechanotransduction axis that converts mechanical stress signals into fibrotic remodeling (41). Under pressure overload, the Hippo kinase cascade is inhibited, leading to dephosphorylation and nuclear translocation of YAP and TAZ, which drive profibrotic gene transcription in cardiac fibroblasts. The ratio of phosphorylated YAP to total YAP in peripheral blood mononuclear cells reflects intracellular pathway activity, offering unique diagnostic specificity for mechanical overload-driven interstitial fibrosis (42). Notably, YAP can also bind directly to Smad proteins, amplifying TGF-*β*-mediated fibrotic responses and forming a key crosstalk node between mechanical stress and humoral profibrotic signals. Together with the TGF-*β* cascade, these three signaling pathways form a multi-dimensional regulatory network of myocardial fibrosis. Each pathway is preferentially activated by specific pathological triggers—mechanical stress, metabolic injury, or ischemic damage. Their corresponding pathway-specific biomarkers complement traditional pan-fibrosis markers, enabling more precise etiological subtyping and activity assessment.

The renin-angiotensin-aldosterone system (RAAS) and neurohumoral pathways, such as endothelin-1, play crucial roles in myocardial fibrosis by promoting fibroblast activation, proliferation, and collagen synthesis. In hypertensive heart disease, prolonged angiotensin II (Ang II) stimulation is a primary factor driving myocardial remodeling and fibrosis (43). Research shows that sacubitril/valsartan (LCZ696), an angiotensin receptor-neuropeptidase inhibitor, can reduce myocardial fibrosis and cardiac remodeling post-myocardial infarction by inhibiting the Wnt/*β*-catenin signaling pathway and increasing sFRP-1 expression (44). This highlights the potential of targeting the RAAS pathway for anti-fibrotic therapy. Inflammatory and immune responses are essential in the onset and maintenance of fibrosis. Various inflammatory cytokines, particularly those in the interleukin family, are closely linked to fibrosis. However, they differ significantly in whether they exert profibrotic effects directly on cardiac fibroblasts or indirectly through immune cell recruitment (45). This mechanistic distinction determines the specificity of these cytokines as biomarkers for fibroblast-driven fibrosis. Interleukin-11 (IL-11) primarily exerts its profibrotic effects through direct action on cardiac fibroblasts. In stress-induced myocardial fibrosis models, IL-11 signaling is significantly upregulated in resident cardiac fibroblasts (46). The binding of IL-11 to its receptor IL-11R*α* directly activates intracellular STAT3 and ERK pathways, promoting fibroblast-to-myofibroblast transition and excessive collagen synthesis. Treatment with anti-IL-11 antibodies significantly reduces pro-fibrotic gene expression and myocardial collagen deposition. This anti-fibrotic effect remains effective in T-cell- and macrophage-depleted animal models, confirming its independence from secondary immune cell recruitment (47). Due to this direct fibroblast-targeting mechanism, circulating IL-11 levels accurately reflect the intrinsic activation state of cardiac fibroblasts, showing high specificity for fibroblast-driven interstitial fibrosis. In contrast, cytokines from the IL-17 family mediate myocardial fibrosis primarily through indirect immune-mediated pathways, with minimal direct effects on fibroblasts (48). IL-17 is mainly secreted by Th17 cells, and its core profibrotic mechanism involves recruiting monocytes, macrophages, and neutrophils to the myocardial interstitium. These cells then release TGF-*β*, TNF-*α*, and other secondary fibrogenic mediators, indirectly driving fibroblast activation (49). Although IL-17 can bind to receptors on cardiac fibroblasts and exert mild direct stimulatory effects, this contribution is negligible compared to its immune-recruitment effect. From a biomarker perspective, circulating IL-17 levels mainly reflect systemic T-cell-mediated inflammatory activity rather than the intrinsic activation level of cardiac fibroblasts (50). Therefore, IL-17 has limited specificity for quantifying fibroblast-driven fibrosis and is more suitable as an auxiliary indicator of inflammatory myocardial injury.

These fibrogenic signaling pathways are interconnected, forming complex crosstalk and feedback loops that lead to non-linear biomarker responses during disease progression (51). At the core, TGF-*β* signaling interacts intricately with the RAAS and Wnt/*β*-catenin pathways. Angiotensin II not only increases TGF-*β* transcription in cardiac fibroblasts and endothelial cells but also enhances Smad2/3 phosphorylation through AT1 receptor-mediated activation of MAPK and PI3K/Akt co-signaling, thereby amplifying the profibrotic response. In return, activated TGF-*β* signaling upregulates AT1 receptor expression on fibroblast membranes, creating a positive feedback loop that accelerates fibrogenesis once a specific activation threshold is reached (52). Similarly, the TGF-*β* and Wnt/*β*-catenin pathways synergize through the physical interaction between Smad3 and *β*-catenin, forming a transcriptional complex that cooperatively activates downstream profibrotic genes such as CTGF and *α*-SMA (53). This interaction results in a super-additive effect on collagen synthesis, which cannot be explained by simply adding the activities of individual pathways.

Inflammatory and fibrogenic pathways are interconnected through bidirectional feedback loops. Proinflammatory cytokines like IL-11 and IL-17 activate cardiac fibroblasts to secrete extracellular matrix components (54). In response, these activated fibroblasts release chemokines such as MCP-1, which recruit macrophages and T cells to the interstitium. This recruitment sustains local inflammation, creating a vicious cycle of inflammation and fibrosis. This positive feedback loop accounts for the rapid and non-linear increase in fibrosis biomarkers once the condition progresses from subclinical interstitial fibrosis to overt reactive fibrosis (55). Conversely, endogenous negative regulatory loops, such as Smad7-mediated ubiquitination degradation of TGF-*β* receptors and BMP-7-mediated antagonism of Smad signaling, also exist (56). These inhibitory mechanisms result in a plateau phase of biomarker levels during the slow progression of chronic fibrosis. This creates a non-linear dynamic trajectory characterized by alternating periods of rapid increase and relative stagnation. For interpreting circulating biomarkers, these pathway interactions and feedback effects imply that biomarker levels do not correlate linearly with the degree of histological fibrosis. The same biomarker concentration may indicate different disease stages, depending on whether the feedback loop is active (57). Additionally, the rate of biomarker change over time cannot be linearly extrapolated. Understanding these network interactions is crucial for accurately interpreting dynamic longitudinal changes in fibrosis biomarkers.

Recent phase II clinical studies conducted after 2022 highlight variability in the effectiveness of RAAS inhibitors in reducing classic fibrosis biomarkers like Gal-3 and thrombospondin-2 (58). These inhibitors show significant regulatory effects in patients with heart failure with preserved ejection fraction (HFpEF) but are less effective in those with dilated cardiomyopathy. In hypertensive myocardial fibrosis, chronic mechanical pressure overload initiates a self-amplifying RAAS-Wnt/*β*-catenin positive feedback loop, serving as the core fibrogenic cascade (59). Sustained ventricular wall stretch upregulates the local cardiac renin-angiotensin system, leading to abundant angiotensin II production by cardiac fibroblasts and endothelial cells. Mechanical stress and Ang II synergistically promote the secretion of canonical Wnt ligands while suppressing endogenous Wnt antagonists (sFRP-1, Dkk-1) in cardiomyocytes and fibroblasts, amplifying profibrotic signaling (60). Here, inflammatory cell infiltration is a secondary, late-stage event, making inflammatory cytokine pathways a minor contributor to fibrosis progression. Conversely, non-ischemic dilated cardiomyopathy often stems from autoimmune myocardial injury, viral myocarditis sequelae, or genetic cardiomyopathy with chronic immune activation. Its core pathological feature is persistent interstitial infiltration of CD4+ T cells, M1 macrophages, and plasma cells, which secrete large quantities of IL-11, IL-17, and other pro-inflammatory profibrotic cytokines, directly driving fibroblast activation and collagen production (61). In this scenario, the RAAS system is activated secondarily as a compensatory response to ventricular dilation and hemodynamic overload, rather than as the primary driver of fibrogenesis (62). Chronic inflammatory stimulation also downregulates AT1 receptor expression on cardiac fibroblasts, weakening the profibrotic contribution of the RAAS-Wnt axis and making inflammatory cytokine pathways the dominant pathogenic drivers. This heterogeneous response is explained by three interrelated factors with direct implications for precision patient subtyping. First, the differential expression of angiotensin II type 1 (AT1) receptors on cardiac fibroblasts determines the magnitude of direct drug action (63). In HFpEF, primarily caused by chronic pressure overload and hypertension, cardiac fibroblasts maintain high AT1 receptor density, allowing RAAS inhibitors to effectively block Ang II-mediated fibroblast activation and subsequent secretion of Gal-3 and TSP-2 (64). In contrast, in non-ischemic dilated cardiomyopathy (DCM), sustained inflammatory stimulation downregulates fibroblast AT1 receptor expression, reducing the effectiveness of RAAS inhibitors on fibrosis biomarkers. Second, disparate downstream signaling crosstalk across heart failure phenotypes drives divergent biomarker responses (65). In hypertensive HFpEF, myocardial fibrosis is driven by the RAAS-Wnt/*β*-catenin axis: Gal-3 secretion from cardiac macrophages is regulated by Ang II-mediated inflammatory signaling, and TSP-2 transcription is partially controlled by Ang II downstream cascades. This tight pathway coupling allows RAAS inhibition to significantly reduce circulating biomarkers (66). However, in DCM, fibrogenesis is dominated by TGF-*β*/Smad and IL-11/IL-17 inflammatory cytokine pathways, which have limited interaction with the RAAS cascade. This decoupling explains why RAAS inhibitors have minimal effects on circulating Gal-3 and TSP-2 levels in DCM patients, despite their hemodynamic benefits. Third, pharmacodynamic factors amplify the efficacy gap. Advanced-stage DCM patients often present with pronounced neurohormonal activation, impaired renal function, and expanded extracellular volume. These changes alter drug distribution and reduce target tissue drug exposure, further diminishing the biomarker response to standard-dose RAAS inhibitors. Clinically, this mechanistic heterogeneity supports pathway-based patient subtyping. RAAS inhibitors are indicated for patients with RAAS-driven fibrotic phenotypes, such as hypertensive HFpEF, for both hemodynamic control and anti-fibrotic efficacy. In contrast, patients with inflammatory-dominant fibrosis, such as idiopathic DCM, require alternative anti-fibrotic regimens targeting inflammatory cytokine pathways to achieve meaningful biomarker reduction.

From a biomarker discovery standpoint, these well-defined mechanistic cascades can be translated into three specific hypotheses to enhance diagnostic specificity (67). First, pathway-specific cleavage products emerge as promising candidate biomarkers. For instance, the N-terminal fragment of latent TGF-*β* binding protein 1 (LTBP-1), released during latent TGF-*β* activation, and the soluble ectodomain of interleukin-11 receptor *α* (IL-11R*α*), shed during inflammatory fibrotic signaling, can serve as pathway-specific surrogate markers instead of non-specific total collagen metabolites. Second, post-translationally modified circulating proteins can differentiate cardiac-specific fibrotic activity from systemic collagen turnover (68). Phosphorylated Smad2/3, carried by platelet-derived extracellular vesicles, and hydroxylated or glycosylated type I collagen neo-epitopes, generated during cardiac matrix cross-linking, demonstrate higher tissue specificity than unmodified full-length collagen peptides (69). Third, upstream signaling regulators with cardiac-enriched expression, such as UII and YTHDF1, can be developed as pathway activation markers. These markers can identify the dominant profibrotic signaling axis in individual patients, aiding in the design of etiology-specific biomarker panels.

### 3.2 Standardized taxonomy and evaluation criteria for myocardial fibrosis biomarkers

#### 3.2.1 Taxonomy rationale and framework

The traditional classification of myocardial fibrosis biomarkers often combines molecular origin, carrier status, and detection methods without a unified standard, creating confusion, particularly for emerging categories like extracellular vesicles (EVs) and exosomal miRNAs (70). To address this issue, we propose a two-level standardized taxonomy based on molecular origin, carrier status, and measurement modality (71). At the primary level, biomarkers are categorized into two main modalities: circulating liquid-biopsy biomarkers and *in vivo* imaging biomarkers. The circulating category is further divided into three subclasses, determined by whether the molecule is encapsulated by a lipid bilayer and the associated sample pretreatment process (72). This framework provides clear differentiation criteria and consistent operational definitions across all classes, aligning with the chapter structure of this review.

#### 3.2.2 Detailed classification with operational definitions

##### 3.2.2.1 Class 1: circulating soluble protein and peptide biomarkers

Operational Definition: Unencapsulated, freely soluble proteins or peptides in peripheral blood are released directly into circulation during myocardial extracellular matrix (ECM) synthesis, degradation, or cellular stress responses (73). These molecules indicate systemic or local fibrotic remodeling activity. Core Differentiation Criteria: These proteins lack lipid bilayer encapsulation and can be detected directly in whole serum or plasma without particle separation. Their molecular weight ranges from small peptide fragments (∼10 kDa) to large matricellular proteins (∼100 kDa) (74). Primary Detection Techniques: Detection methods include enzyme-linked immunosorbent assay (ELISA), chemiluminescent immunoassay (CLIA), immunoturbidimetry, and targeted proteomics. Representative Biomarkers: Key biomarkers include collagen metabolism markers such as PIIINP, PICP, and MMPs/TIMPs, as well as inflammatory and matricellular proteins like Galectin-3, thrombospondin-2, GDF-15, and periostin (75). Clinical Translation Status: This is the most clinically advanced class, with several commercial detection kits available and guideline recommendations in place.

##### 3.2.2.2 Class 2: circulating cell-free nucleic acid biomarkers

**Operational Definition:** Non-vesicle-encapsulated nucleic acid fragments are passively released into peripheral blood from damaged, apoptotic, or activated cardiac cells (76). These fragments influence epigenetic regulation of fibrotic signaling pathways and act as early molecular indicators of subclinical fibrosis. **Core Differentiation Criteria:** These nucleic acid fragments are free in plasma, lacking lipid membrane protection, and are vulnerable to RNase degradation in circulation. They are extracted directly from total plasma nucleic acids without prior particle enrichment. **Primary Detection Techniques:** The main methods for detecting these fragments include quantitative real-time PCR (qRT-PCR), microarray hybridization, and next-generation sequencing (NGS) (77). **Representative Biomarkers:** Notable biomarkers include circulating microRNAs, such as miR-21 and miR-133a, and circular RNAs like circPAN3 (78). **Clinical Translation Status:** These biomarkers are currently in the multicenter clinical validation phase. However, their clinical application is limited by high detection costs and the absence of standardized quantification methods (79).

##### 3.2.2.3 Class 3: extracellular vesicle (EV)-derived biomarkers

Operational Definition: Protein, mRNA, or non-coding RNA molecules are encapsulated within lipid bilayer membrane vesicles actively secreted by cardiomyocytes, cardiac fibroblasts, and endothelial cells (80). Exosomes, measuring 30–150 nm in diameter, are the most extensively studied subclass, with exosomal miRNAs emerging as key markers in this category (81). Core Differentiation Criteria: These molecules are protected by a lipid bilayer membrane, providing greater biological stability than cell-free nucleic acids. They require specific isolation steps, such as ultracentrifugation, size-exclusion chromatography, or affinity purification, before detecting target molecules. This requirement distinguishes them from cell-free nucleic acid biomarkers. Primary Detection Techniques: Detection involves EV isolation combined with qRT-PCR, flow cytometry, nanoparticle tracking analysis (NTA), and EV proteomics. Representative Biomarkers: Notable examples include exosomal miR-21, exosomal miR-29, and fibroblast-derived EV surface proteins. Clinical Translation Status: Since 2022, this research field has been rapidly expanding. These biomarkers offer superior tissue specificity compared to free nucleic acids (82). However, the lack of standardized isolation protocols remains a significant hurdle for clinical translation.

##### 3.2.2.4 Class 4: cardiac magnetic resonance (CMR) imaging biomarkers

Operational Definition: Quantitative *in vivo* imaging parameters obtained through cardiac magnetic resonance (CMR) scanning reflect myocardial tissue microstructure, extracellular matrix (ECM) composition, and mechanical properties linked to fibrotic remodeling (83). Core Differentiation Criteria: These parameters allow for non-invasive *in vivo* measurement with precise spatial anatomical localization. They can differentiate between focal and diffuse fibrosis and are detected using MRI scanners equipped with specialized post-processing software, eliminating the need for blood sampling (84). Primary Detection Techniques: The main techniques include native T1 mapping, extracellular volume fraction (ECV), late gadolinium enhancement (LGE), and CMR feature tracking (CMR-FT). Representative Biomarkers: Key biomarkers include native T1 value, ECV, LGE extent, and global longitudinal strain (GLS) (85). Clinical Translation Status: This method is recognized as the non-invasive gold standard for assessing myocardial fibrosis. Since 2022, AI-assisted quantification has enhanced its clinical accessibility (86).

#### 3.2.3 Performance evaluation criteria and cross-category comparison

Traditionally, biomarker evaluation focused on diagnostic sensitivity and specificity (87). However, since 2022, this system has broadened to include effect size, cross-population stability, dynamic monitoring capability, treatment response correlation, and clinical cost-effectiveness. These additions have been validated by large cohort studies and meta-analyses. A systematic comparison across categories, based on this expanded framework, is summarized as follows: Circulating soluble protein markers, such as PICP, PIIINP, and Gal-3, offer moderate diagnostic efficacy with an AUC ranging from 0.65 to 0.78 (88). Their low detection cost and ease of sampling make them suitable for primary population screening. However, their main drawback is poor organ specificity, as levels can be easily influenced by liver or renal dysfunction and systemic inflammation. CMR imaging biomarkers, including ECV and optimized T1 mapping, demonstrate the highest diagnostic effect size with an AUC greater than 0.85 (89). These biomarkers maintain stable performance across diverse populations and are considered the reference standard for non-invasive fibrosis quantification. Additionally, AI enhancements have further reduced inter-observer variability. Cell-free nucleic acid and EV-derived markers exhibit high tissue specificity and ultra-early diagnostic potential (90). While EV-encapsulated molecules offer better biological stability than free nucleic acids, they require more complex sample preparation. Most panels are still in the clinical verification phase, and high testing costs limit their routine clinical use (91). In conclusion, the ideal biomarker panel should integrate high diagnostic accuracy, dynamic monitoring capability, and standardized detection protocols to optimize clinical utility.

### 4.1 Markers of collagen synthesis: pro-III-N-terminal peptide (PIIINP) and pro-I-C-terminal peptide (PICP)

Pro-III-N-terminal peptide (PIIINP) is a fragment released into the bloodstream during type III collagen synthesis. Elevated serum PIIINP levels often indicate active type III collagen synthesis in the cardiac interstitium, serving as a potential marker for early and active myocardial fibrosis. After an acute myocardial infarction (AMI), serum PIIINP levels are closely linked to the onset and progression of myocardial fibrosis (92, 93). Research indicates that in patients with ST-segment elevation myocardial infarction (STEMI) and preserved left ventricular ejection fraction, a serum PIIINP level of ≥381.4 ng/mL on day 12 post-infarction is an independent risk factor for developing myocardial fibrosis within a year (92). This threshold value (381.4 ng/mL) and corresponding unit are directly extracted from the original cited cohort research without unit conversion. The discrepancy between this cutoff and lower PIIINP cutoffs widely reported in other literature is mainly attributed to differences in study populations (only STEMI patients with preserved LVEF were enrolled in the source trial), blood sampling timing (12 days after acute myocardial infarction, a peak window of collagen synthesis after ischemic injury), and detection reagent kits with distinct calibration standards adopted in the original study (94). PIIINP exhibits a distinct temporal profile following acute myocardial injury: serum levels begin to rise 3–5 days after ischemic insult, peak at days 10–14 during active type III collagen synthesis, and decline rapidly thereafter with an estimated circulating half-life of 5–7 days. This kinetic pattern implies that single-timepoint PIIINP values must be interpreted in the context of time since injury: values during the 10–14 day peak reflect maximal acute fibrotic activity, while those outside this window may underestimate ongoing fibrosis. Monitoring PIIINP during hospitalization could help identify patients at high risk for myocardial fibrosis early on. In patients with acute decompensated heart failure (ADHF), PIIINP serves as an independent prognostic factor for all-cause mortality (95). When used alongside NT-proBNP, it provides additional prognostic value beyond NT-proBNP alone (96). However, in elderly patients (≥75 years old) with chronic kidney disease, the correlation between PIIINP and myocardial fibrosis severity diminishes, reducing its positive predictive value from 76% to 42%. This loss of diagnostic accuracy can be explained by three interrelated pathophysiological mechanisms: First, impaired renal clearance distorts circulating PIIINP concentrations. PIIINP is a small-molecule peptide (∼45 kDa) predominantly eliminated via glomerular filtration. As estimated glomerular filtration rate declines, PIIINP accumulates passively in the circulation, independent of cardiac collagen synthesis rate, breaking the linear correlation between serum levels and myocardial fibrotic activity. In comparison, PICP has a larger molecular weight (∼100 kDa) and is less dependent on renal clearance, thus retaining partial diagnostic value in moderate renal insufficiency (97). Second, hepatic dysfunction introduces dual interference. PICP is primarily metabolized and cleared via mannose receptor-mediated endocytosis by hepatic sinusoidal endothelial cells; impaired liver function directly reduces its clearance rate and elevates baseline circulating levels (98). Meanwhile, liver cirrhosis itself is accompanied by massive systemic collagen synthesis, which further increases extracardiac input of both PICP and PIIINP. This combined effect of reduced clearance and elevated extracardiac production masks cardiac-derived fibrotic signals and severely compromises diagnostic specificity (99). Third, age-related systemic collagen turnover remodeling further reduces the signal-to-noise ratio. Advanced age independently drives generalized connective tissue remodeling in skin, vascular walls, and skeletal tissue, altering whole-body collagen synthesis and degradation rates and raising baseline circulating levels of collagen precursor peptides (100). This elevates the systemic background signal independent of cardiac pathology, diluting the relative contribution of myocardial-derived PIIINP. When superimposed on subclinical renal and hepatic functional decline common in elderly populations, this effect completely abolishes the correlation between circulating markers and cardiac fibrosis severity (101). Quantitative effect size analysis shows that PIIINP has a strong prognostic effect in young and middle-aged patients with a single cardiovascular disease (HR = 1.87, 95%CI:1.42–2.46), but its predictive power significantly weakens in patients with multiple comorbidities (HR = 1.13, 95%CI:0.89–1.44). This indicates that PIIINP is suitable only for specific populations, limiting its clinical application (102). It represents an incremental exploration of traditional markers rather than an original innovation. From a pathophysiological perspective, PIIINP is a marker of active, reversible fibrotic processes: it is released during *de novo* type III collagen synthesis, which predominates in the early, dynamic phase of fibrosis when interstitial remodeling is still responsive to anti-fibrotic interventions. It does not reflect the burden of already formed, irreversible scar tissue.

Type I procollagen C-terminal peptide (PICP) is a fragment released during the synthesis of type I collagen, a vital component of mature cardiac scar tissue and stable fibrosis. Elevated serum PICP levels often signify advanced fibrosis. In patients with hypertrophic cardiomyopathy (HCM), plasma PICP levels are independently linked to adverse cardiovascular outcomes. Research on hypertrophic obstructive cardiomyopathy (HOCM) patients found that the plasma PICP-to-type I collagen C-terminal cross-linked peptide (ICTP) ratio (PICP/ICTP) is a strong prognostic marker for predicting new-onset atrial fibrillation and heart failure. In non-ischemic dilated cardiomyopathy (NIDCM), PICP levels positively correlate with late gadolinium enhancement (LGE) observed via cardiac magnetic resonance imaging (CMR) and can independently predict adverse cardiovascular outcomes (103). Additionally, in heart failure patients, baseline PICP levels can predict left ventricular reverse remodeling (LVRR) and clinical outcomes. Patients with low PICP levels (<108.1 ng/mL) experience more significant LVRR and a reduced risk of heart failure-related events, especially in non-ischemic heart failure (98). These studies collectively highlight the importance of PICP as a marker for assessing post-myocardial infarction remodeling, fibrotic burden in hypertrophic cardiomyopathy, and overall prognosis. Notably, PICP is more effective for evaluating chronic stable fibrosis, while PIIINP better reflects acute active fibrosis. Kinetic differences further support this distinction: after acute myocardial injury, PICP levels rise with a delayed onset, peaking at 4–6 weeks post-infarction, and decline slowly with a circulating half-life of 15–20 days, reflecting the gradual deposition and maturation of type I collagen in stable scar tissue. The combined detection of both markers enhances diagnostic efficacy (AUC increased by 0.07–0.10 compared to single detection). Importantly, their differing kinetic profiles allow clinicians to distinguish active fibrotic injury from established stable fibrosis, even with single timepoint measurements, when interpreted in the clinical context: in patients within 2 weeks of acute cardiac injury, elevated PIIINP with normal or mildly elevated PICP indicates active, potentially reversible fibrotic injury; in patients 3 months or later after the inciting event, persistently elevated PICP with normalized PIIINP indicates established, stable scar fibrosis with low reversibility. When combined with TGF-*β* pathway mechanisms, changes in the PICP/PIIINP ratio correspond to the switching of fibrotic phenotypes mediated by Smad protein subtypes, explaining the ratio's unique value in distinguishing different fibrosis stages. In contrast to PIIINP, PICP primarily reflects the synthesis of type I collagen, the main component of mature, cross-linked scar tissue. Elevated PICP indicates progression toward stable, largely irreversible fibrosis, making it more suitable for assessing established disease burden and long-term prognosis rather than detecting early reversible activity.

PIIINP and PICP are key indicators of type III and type I collagen synthesis, respectively, during different fibrosis stages—active and stable. Assessing both markers together provides a comprehensive view of the active stage and nature of myocardial fibrosis. For instance, in patients with type 2 diabetes and heart failure with varying ejection fractions, the PICP/PIIINP ratio is elevated in those with heart failure with mid-range ejection fraction (HFmrEF) compared to those with heart failure with preserved ejection fraction (HFpEF). This suggests a potential shift toward type I collagen synthesis in HFmrEF patients. However, interpreting these markers requires careful consideration of the clinical context. Factors like age, renal function impairment, and liver disease can affect marker levels through altered clearance and systemic collagen turnover. This necessitates population-stratified cutoff values and, when feasible, correction formulas for hepatic and renal function. For elderly patients and those with multiple comorbidities, combining collagen metabolites with cardiac tissue-specific markers (e.g., thrombospondin-2) is advised to reduce systemic interference and enhance diagnostic accuracy.

### 4.2 Markers of collagen degradation and regulation: matrix metalloproteinases and their tissue inhibitors (MMPs/TIMPs)

Matrix metalloproteinases (MMPs) and their tissue inhibitors (TIMPs) are crucial in regulating the balance between extracellular matrix (ECM) degradation and deposition, significantly impacting myocardial fibrosis development. MMPs, zinc-dependent endopeptidases, degrade various ECM components, including collagen (104). In cardiac diseases, specific MMP subtypes contribute to collagen breakdown. For instance, MMP-2 is a potential biomarker for early-stage fibrosis in diabetic cardiomyopathy, with its upregulation closely linked to collagen proliferation (105). Elevated MMP-9 levels in heart failure patients are associated with myocardial fibrosis and left ventricular remodeling (106). Additionally, MMP-7 and MMP-10 are implicated in cardiac fibrosis (107, 108). Changes in MMP activity or levels indicate active myocardial matrix remodeling. TIMPs naturally inhibit MMPs by binding to zinc ions at MMP active sites, suppressing their enzymatic activity and promoting ECM deposition (104). In conditions like atrial fibrillation, diabetic cardiomyopathy, and stress-induced heart failure, increased TIMP-1 expression is vital for driving matrix deposition and fibrosis.TIMP-2 and TIMP-4 also regulate MMP activity, collectively influencing the ECM's metabolic balance (109–112). Therefore, the dynamic equilibrium of the MMP/TIMP system is crucial for determining collagen degradation and synthesis rates.

The interplay between matrix metalloproteinases (MMPs) and tissue inhibitors of metalloproteinases (TIMPs) is crucial, with the MMP-9/TIMP-1 ratio serving as a vital marker of the balance between matrix degradation and deposition. In heart failure patients, an imbalance in this ratio is closely linked to poor outcomes. Research indicates that in heart failure with preserved ejection fraction, the activities of MMP-2 and MMP-9, along with alterations in TIMP-1 protein levels, collectively impact extracellular matrix (ECM) remodeling (104). In atrial fibrillation models, miR-146b-5p disrupts the TIMP4/MMP9 balance by inhibiting TIMP4, which leads to increased collagen synthesis and atrial fibrosis (109). This highlights that an imbalance in the MMP/TIMP ratio directly contributes to fibrosis. However, clinical studies yield mixed results regarding the prognostic value of individual MMP/TIMP indicators (113). While some cohorts find the MMP-9/TIMP-1 ratio to be a reliable prognostic marker, studies in diabetic cardiomyopathy cohorts suggest that systemic metabolic disorders can disrupt this ratio, leading to inconsistent results. The predictive power of a single MMP or TIMP subtype is generally weak (AUC < 0.70), and the complex interactions among multiple subtypes hinder the establishment of a unified diagnostic standard (114). Regarding therapeutic response monitoring, evidence from prospective intervention trials shows that in HFpEF patients treated with sacubitril/valsartan, circulating TIMP-1 levels decrease significantly by 12%–18% within 12 weeks of treatment initiation. This reduction precedes improvements in left ventricular global longitudinal strain, suggesting that TIMP-1 downregulation reflects early ECM remodeling before functional recovery is detectable. Conversely, MMP-9 activity does not show consistent changes within the first 8 weeks of mineralocorticoid receptor antagonist (MRA) therapy, but its late increase at 6 months correlates with reverse remodeling of left ventricular volume. This indicates distinct temporal patterns of different MMP/TIMP components during anti-fibrotic treatment. Most research on MMPs and TIMPs focuses on regulatory mechanisms and population applicability, representing incremental progress. Functionally, the MMP/TIMP system reflects the dynamic balance between ECM degradation and deposition, capturing active tissue remodeling processes rather than static scar burden. Changes in this ratio occur before measurable scar accumulation, suggesting these markers as potential indicators of reversible fibrogenic activity, though with limited specificity for cardiac tissue.

### 4.3 Comparative summary of circulating fibrosis biomarkers

To systematically compare the diagnostic and prognostic performance of commonly used circulating myocardial fibrosis markers, we compiled their quantitative diagnostic metrics, advantages, disadvantages, and clinical applicability into a comprehensive comparative table (Table 1). The AUC, sensitivity, and specificity values were sourced from multicenter cohort studies published between 2022 and 2026, as discussed in this narrative review. This table serves as a clear quantitative resource for clinicians to choose suitable biomarkers for risk stratification and therapeutic monitoring.

**Table 1:** Comparative diagnostic and prognostic performance of core circulating myocardial fibrosis biomarkers.

Biomarker	Typical AUC range	Sensitivity/Specificity	Core advantages	Main limitations	Current clinical applicability
PIIINP (Type III collagen marker)	0.67–0.76	Sen:72%–78%; Spe:69%–75%	Reflects early/active collagen synthesis; low testing cost; widely commercialized kits	Poor organ specificity; falsely elevated in renal/liver fibrosis; unstable predictive value in elderly multi-comorbidity populations	Primary screening for STEMI post-infarction early fibrosis; single cardiovascular disease young/middle-aged patients
PICP (Type I collagen marker)	0.66–0.75	Sen:70%–76%; Spe:68%–74%	Represents stable late-stage fibrosis; predicts left ventricular reverse remodeling	Interfered by bone/hepatic collagen metabolism; cannot reflect dynamic fibrotic activity	Long-term prognosis monitoring for hypertrophic cardiomyopathy and non-ischemic heart failure
MMP-9/TIMP-1 ratio	0.63–0.69	Sen:65%–71%; Spe:64%–70%	Directly reflects ECM synthesis-degradation balance; suitable for atrial fibrosis assessment	Result interference from systemic metabolic disorders; inconsistent cross-cohort results	Auxiliary index for atrial fibrillation related myocardial remodeling
Galectin-3 (Gal-3)	0.68–0.77	Sen:74%–79%; Spe:70%–76%	Captures inflammatory-fibrotic crosstalk; recommended Class IIa by heart failure guidelines	Affected by systemic inflammation and renal dysfunction; minimal change under anti-fibrotic drugs	Risk stratification for HFpEF and chronic Chagas cardiomyopathy
Thrombospondin-2 (TSP-2)	0.72–0.78	Sen:76%–81%; Spe:73%–79%	Cardiac tissue-biased signal; nearly unaffected by aging and kidney injury; superior cardiovascular mortality HR	No obvious dynamic fluctuation after SGLT2i/ARNI intervention; cannot monitor anti-fibrotic treatment response	Long-term adverse cardiovascular event prediction for CRT candidates and hypertensive heart disease
GDF-15	0.69–0.75	Sen:75%–80%; Spe:67%–73%	Pan-myocardial stress marker; correlates with NYHA grading	Non-fibrosis-specific; rises in multiple organ injury	Combined use with collagen markers for comprehensive myocardial injury evaluation
Periostin	0.74–0.80	Sen:78%–83%; Spe:75%–81%	High subclinical fibrosis predictive ability	Insufficient large-sample external validation; no unified clinical cutoff values	Exploratory screening for early asymptomatic myocardial fibrosis
Circulating non-coding RNA (miR-21/circPAN3 panel)	0.82–0.87	Sen:84%–89%; Spe:82%–87%	Highest tissue specificity; ultra-early fibrotic molecular signal	High detection cost; lack uniform standardized testing protocols	Preclinical research and small-cohort early diagnosis trials, not routine clinical use

Traditional collagen metabolites, such as PIIINP and PICP, along with inflammatory proteins like Gal-3 and TSP-2, demonstrate moderate diagnostic efficacy, with AUC values ranging from 0.66 to 0.78. These proteins benefit from mature detection systems that are well-suited for routine outpatient screening. However, their utility is limited by poor organ specificity. In contrast, the novel single protein periostin shows a slightly higher AUC, but the lack of standardized cutoffs and multicenter verification remains a challenge. Non-coding RNA panels offer superior diagnostic performance for detecting subclinical fibrosis, with AUC values exceeding 0.80. Despite their effectiveness, the high cost and lack of standardized detection methods hinder their widespread clinical adoption. Among conventional circulating markers, TSP-2 is particularly noteworthy due to its minimal interference from renal function and age, making it the most reliable single marker for long-term prognosis assessment.

## 5 Novel circulating proteins and peptide biomarkers

### 5.1 Galectin-3 (Gal-3) and thrombospondin-2

**Figure 2:**
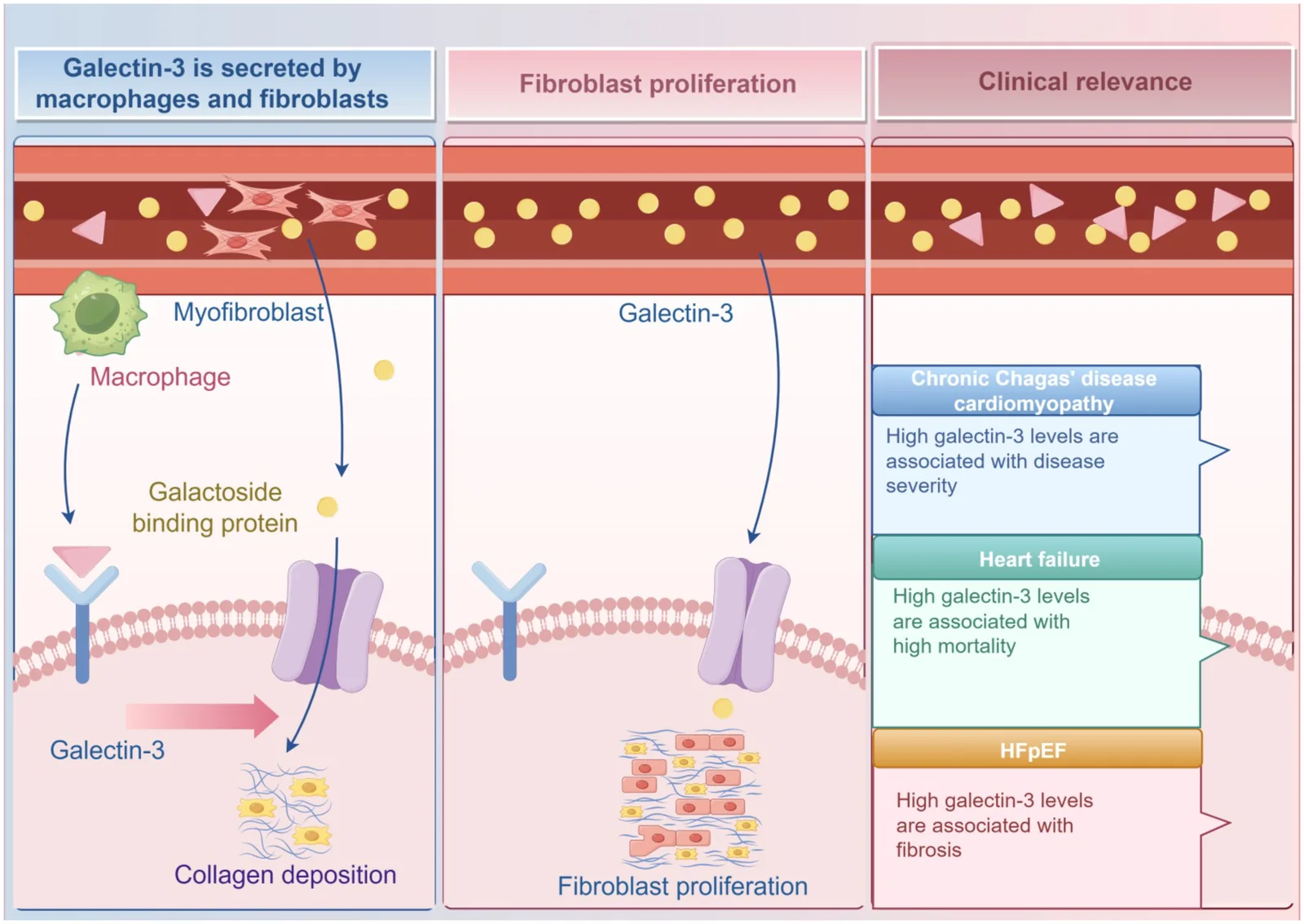
Galectin-3 (Gal-3) is a *β*-galactosidase-binding protein secreted by activated macrophages and myofibroblasts, playing a key role in fibrosis. It promotes fibroblast proliferation, collagen deposition, and inflammatory responses. In chronic Chagas cardiomyopathy, elevated Gal-3 levels are significantly associated with disease severity. Similarly, in both acute and chronic heart failure, high Gal-3 levels correlate with increased mortality rates. Furthermore, in patients with left ventricular non-compaction and heart failure with preserved ejection fraction (HFpEF), elevated Gal-3 levels are strongly linked to extensive fibrosis.

Galectin-3 (Gal-3), a *β*-galactosidase-binding protein secreted by activated macrophages and myofibroblasts, is crucial in the development of cardiac fibrosis (115). It promotes fibroblast proliferation, collagen deposition, and inflammatory responses, thereby facilitating fibrosis (116). In heart failure (HF) pathophysiology, Gal-3 is vital due to its roles in cardiac fibrosis, inflammation, and ventricular remodeling (117). For instance, in chronic Chagas cardiomyopathy patients, elevated Gal-3 levels significantly correlate with disease severity (118). Gal-3 is particularly associated with heart failure with preserved ejection fraction (HFpEF). In patients with left ventricular non-compaction and HFpEF, Gal-3 levels are notably high and closely linked to extensive fibrosis (119). Despite its potential as a biomarker, Gal-3's utility is limited by its biological complexity. Its levels are affected by renal function and systemic inflammatory status, complicating its reliability as a biomarker (115). (Figure 2).

Thrombospondin-2 (TSP-2) is a secreted matricellular protein in the thrombospondin superfamily (120). Its primary profibrotic mechanism involves modulating TGF-*β* bioavailability and extracellular matrix cross-linking. TSP-2 activates latent TGF-*β* stored in the cardiac interstitium, which enhances fibroblast activation and collagen deposition, leading to myocardial interstitial fibrosis. Elevated levels of TSP-2 are found in both acute decompensated and chronic heart failure patients. Notably, its circulating concentration remains largely unaffected by aging or renal dysfunction, offering distinct clinical advantages over many other fibrotic markers (121). Studies indicate that TSP-2 is a strong independent predictor of cardiovascular mortality and heart failure-related rehospitalization in heart failure patients. For example, in candidates for cardiac resynchronization therapy (CRT), baseline serum TSP-2 concentrations are closely linked to long-term adverse cardiovascular events (122). Additionally, in hypertensive individuals, TSP-2 levels positively correlate with the left ventricular mass index, underscoring its association with pressure overload-induced ventricular remodeling (123). These clinical attributes highlight the potential of TSP-2 in risk stratification for routine heart failure care.

Galectin-3 (Gal-3) and thrombospondin-2 (TSP-2) are key fibrotic biomarkers included in European and American heart failure guidelines, with Class IIa clinical recommendations. Comparative cohort studies reveal that TSP-2 outperforms Gal-3. Unlike Gal-3, TSP-2 levels remain stable despite renal impairment and systemic inflammation, and it has a higher hazard ratio for cardiovascular death (TSP-2: HR = 2.12; Gal-3: HR = 1.65) (124). In monitoring therapeutic responses, phase II trials of SGLT2 inhibitors and ARNI show that Gal-3 and TSP-2 levels change minimally (<5%) within six months of treatment, with no significant differences between patients with or without left ventricular reverse remodeling. This stability suggests that neither marker is suitable for short-to-medium-term monitoring of anti-fibrotic therapy. Conversely, PIIINP, a collagen synthesis marker, decreases by 15%–22% within 8–12 weeks of eplerenone therapy in post-myocardial infarction patients with systolic dysfunction. The reduction in PIIINP at 12 weeks correlates with improved left ventricular ejection fraction at 12 months, supporting its use as an early predictor of long-term benefits from anti-fibrotic treatment. Mechanistically, Gal-3 reflects systemic inflammatory fibrotic activity, while TSP-2 is linked to cardiac-specific extracellular matrix remodeling, explaining their varied diagnostic effectiveness across patient groups. Even after adjusting for clinical risk factors (age, eGFR, NYHA, LVEF, hs-CRP), both biomarkers independently predict long-term adverse events, with TSP-2 showing a stronger effect (adjusted HR = 1.98 vs. Gal-3 adjusted HR = 1.43). When added to baseline clinical risk models, TSP-2 enhances the model's C-index from 0.62 to 0.73, while Gal-3 increases it to only 0.67, indicating TSP-2 provides more distinct prognostic information beyond conventional metrics. Regarding fibrotic phase reflection, Gal-3 indicates ongoing inflammatory-fibrotic interactions and macrophage-driven fibrogenesis, marking active disease progression. TSP-2, by influencing TGF-*β* bioavailability and extracellular matrix cross-linking, is associated with both active remodeling and scar maturation. Its stable levels make it more suitable for prognostic stratification of established disease rather than monitoring reversible activity. From a kinetic perspective, Gal-3 levels rise quickly within 48 h of acute inflammatory-fibrotic activation and fluctuate with systemic inflammation, making it sensitive to acute disease flares but less stable for chronic risk assessment. In contrast, TSP-2 increases gradually with minimal short-term variability, making it more reliable for assessing chronic fibrotic burden rather than acute injury dynamics. However, multiple phase II SGLT2i trials confirm that neither Gal-3 nor TSP-2 levels significantly change following drug therapy (<5% change), indicating their unsuitability for dynamic monitoring of anti-fibrotic responses. Mechanistically, Gal-3 primarily reflects systemic inflammatory activity, while TSP-2 mirrors cardiac-specific extracellular matrix remodeling, explaining their differing diagnostic performances across patient subgroups (125).

### 5.2 Growth differentiation factor-15 (GDF-15) and other novel molecules

Growth differentiation factor-15 (GDF-15) is a critical stress response factor in various cardiovascular diseases (126). Research indicates that GDF-15 expression rises during myocardial cell stress and injury (127). In patients experiencing acute myocardial infarction (AMI), plasma GDF-15 levels are significantly elevated (128). Although these levels may not differ substantially from those in patients with unstable angina, they are closely linked to myocardial injury (129). Importantly, GDF-15 levels are strongly associated with the progression of myocardial fibrosis. In heart failure patients post-myocardial infarction, serum GDF-15 levels are significantly higher than in healthy individuals and increase with the NYHA functional class, reflecting disease severity (130). This elevation is closely tied to traditional biomarkers of myocardial fibrosis, such as pro-N-terminal pro-collagen type I (PINP), pro-N-terminal pro-collagen type III (PIIINP), and transforming growth factor-*β* (TGF-*β*) (131). Basic research has shed light on the mechanisms by which GDF-15 affects cardiac fibrosis (132). Cardiac fibroblasts naturally express GDF-15, and its expression is further stimulated by the fibrogenic factor endothelin-1 (ET-1). Additionally, administering exogenous GDF-15 directly promotes fibroblast proliferation, highlighting its role in myocardial fibrosis (133). While GDF-15 is not specific to fibrosis, it serves as a valuable indicator of overall myocardial stress, injury, and subsequent fibrotic remodeling (134, 135).

Most studies on GDF-15 focus on validating its correlation with multi-indicator combined models, demonstrating incremental improvements. In contrast, truly novel exploratory molecules such as periostin, Fibroblast Growth Factor-23 (FGF-23), and adiponectin are emerging as promising candidates (136). Initial longitudinal cohort studies have confirmed periostin's predictive value for the progression of subclinical myocardial fibrosis. Research on FGF-23 has revealed its indirect profibrotic mechanism through the renin-angiotensin-aldosterone system (RAAS)–cardiac axis. However, these molecules remain in the early exploratory stage, lacking large-sample external validation and standardized cutoff values. Mechanistically, these novel molecules interact with the TGF-*β* pathway, RAAS, and inflammatory pathways, forming a complex molecular network underlying myocardial fibrosis (137). This interaction also provides a theoretical foundation for integrating multiple biomarkers in future research.

## 6 Cardiac magnetic resonance imaging: the “gold standard” for non-invasive assessment of fibrosis

### 6.1 Native T1 values and extracellular volume fraction (ECV)

Cardiac MRI T1 mapping, particularly through measuring native T1 values and extracellular volume fraction (ECV), has transformed the non-invasive quantitative assessment of myocardial fibrosis. Native T1 values, obtainable without gadolinium contrast, are highly sensitive for detecting myocardial edema and fibrotic changes (138). ECV, derived from T1 values before and after contrast administration, quantitatively reflects the expansion of the myocardial extracellular space. Notably, elevated ECV does not specifically indicate collagen deposition; it can result from three overlapping pathological processes: (1) interstitial collagen fiber deposition, indicating stable structural fibrosis; (2) interstitial edema, due to acute ischemia, inflammation, or reperfusion injury; and (3) inflammatory cell infiltration, signifying active inflammatory fibrogenesis. While all three conditions expand the extracellular compartment and increase ECV values, they differ in reversibility and clinical prognosis. Collagen-dominant ECV elevation represents irreversible structural remodeling and is a strong predictor of long-term adverse cardiovascular events. In contrast, edema- or inflammation-dominant ECV elevation is highly reversible with targeted treatment.

To effectively differentiate the pathological components of extracellular volume (ECV) elevation in clinical practice, a multi-sequence cardiac magnetic resonance (CMR) interpretation strategy is advised. T2 mapping is crucial for identifying interstitial edema, as elevated T2 values confirm the presence of edematous components. Late gadolinium enhancement (LGE) characterizes focal replacement fibrosis. By dynamically monitoring ECV and T2 parameters, clinicians can distinguish active inflammatory infiltration from stable scar tissue. This multi-parameter approach prevents the common misinterpretation of transient interstitial edema as permanent fibrotic scar tissue. When used alongside complementary sequences to confirm collagen-dominant interstitial changes, ECV is widely recognized as the reference standard for non-invasive assessment of diffuse myocardial fibrosis. Research shows that collagen-confirmed ECV strongly correlates with histological fibrosis area, offering exceptional diagnostic value across various diseases. For instance, in hypertrophic cardiomyopathy (HCM) patients, ECV values are significantly elevated even without LGE, primarily indicating diffuse interstitial collagen deposition. Similarly, in chronic conditions like diabetic cardiomyopathy and systemic lupus erythematosus, elevated ECV serves as a sensitive marker of myocardial interstitial involvement, dominated by progressive collagen accumulation. In contrast, during acute myocarditis and the first week following an acute myocardial infarction, ECV elevation typically reflects a combination of edema, inflammatory cell infiltration, and early collagen deposition. In such cases, dynamic re-evaluation is necessary to differentiate reversible injury from permanent fibrosis.

Beyond diagnosis, extracellular volume (ECV) serves as a robust prognostic tool. Numerous studies have established that elevated collagen-dominant ECV is an independent prognostic indicator, identifying high-risk patients with preserved ejection fractions who already show signs of structural myocardial fibrosis. Since 2022, cardiovascular magnetic resonance (CMR) has advanced significantly in three main areas (139). Firstly, technical improvements include the development of supervised deep learning models for fully automatic quantification of native T1 and ECV. These models, typically based on 2D U-Net convolutional neural networks, are trained using expert-manually annotated myocardial contours as the gold standard, eliminating the need for additional manual labeling during clinical inference. This innovation reduces inter-observer variability by over 60% and cuts post-processing time from 30 min to just 2 min. Validation against two board-certified cardiovascular radiologists shows that AI-generated ECV values achieve an intraclass correlation coefficient (ICC) of 0.92, matching the diagnostic accuracy of senior readers. Additionally, optimized rapid scanning sequences now accommodate critically ill patients who cannot hold their breath. Secondly, in model development, a combined serum ferritin–ECV model achieves an area under the curve (AUC) of 0.89 for early diagnosis of post-myocardial infarction myocardial fibrosis, outperforming standalone ECV measurement (AUC = 0.81) and standalone serum ferritin (AUC = 0.74). This diagnostic enhancement arises from the distinct mechanistic information captured by the two biomarkers, rather than mere additive statistical power (140). Pathophysiologically, ECV quantifies the structural expansion of myocardial extracellular space due to collagen deposition, indicating established interstitial remodeling. In contrast, serum ferritin reflects intracellular iron overload and reactive oxygen species (ROS)-mediated fibroblast activation following ischemia-reperfusion injury, a molecular event preceding measurable interstitial collagen accumulation (141). Multivariate logistic regression analysis confirms that both ECV and serum ferritin independently predict post-infarction fibrosis after adjusting for age, infarct size, and renal function, verifying that they provide complementary diagnostic information targeting different stages of fibrogenesis (142). The combined model achieves a net reclassification improvement (NRI) of 0.23 (95% CI: 0.15–0.31) compared to ECV alone: serum ferritin identifies early subclinical fibrosis with normal ECV values, while ECV confirms advanced structural lesions with ambiguous ferritin levels, highlighting the complementary benefits of early molecular sensitivity and structural specificity (143). Lastly, the issue of hematocrit-affected ECV readings has been addressed with standardized correction formulas, significantly enhancing ECV reproducibility in multicenter studies (144). For therapeutic response monitoring, longitudinal cohort studies have characterized the temporal pattern of CMR biomarkers during anti-fibrotic treatment: in hypertensive heart disease patients receiving spironolactone, native T1 values begin to decrease significantly at 6 months, while ECV reduction reaches statistical significance at 12 months, and both imaging changes lag behind the early decline of circulating PIIINP observed at 3 months (145). Notably, the reduction in ECV at 12 months strongly correlates with the improvement in left ventricular diastolic function at 24 months, establishing a temporal sequence where extracellular matrix regression precedes functional recovery. These technical upgrades and model developments in CMR imaging biomarkers represent substantial innovations, translating to clinical practice more rapidly than incremental optimizations of circulating markers. Pathophysiologically, native T1 values and ECV do not solely represent irreversible scar; they reflect the expanded extracellular compartment, which includes both reversibly increased interstitial fluid and active fibroblast proliferation in early fibrosis, as well as accumulated collagen deposition in later stages (146). Longitudinal studies have confirmed that ECV can decrease significantly after effective anti-fibrotic therapy, confirming its ability to capture reversible active fibrotic processes, in addition to reflecting established fibrotic burden.

### 6.2 Late gadolinium enhancement and feature tracking techniques

Late gadolinium enhancement (LGE) is a widely recognized cardiac MRI technique used to evaluate focal replacement fibrosis. It identifies enhancement patterns—subendocardial, transmural, and mid-myocardial—that enable precise localization and characterization of fibrotic regions, such as scar tissue from myocardial infarction. The extent of LGE is closely linked to adverse clinical outcomes. In hypertrophic cardiomyopathy, LGE serves as an independent predictor of non-sustained ventricular tachycardia (147). For patients with primary aldosteronism, LGE significantly correlates with hypertension duration, blood pressure burden, and left ventricular mass index, underscoring pressure-induced myocardial remodeling (148). However, the technique depends on normal myocardium as a reference, limiting its effectiveness in assessing diffuse, non-specific interstitial fibrosis. Importantly, LGE-positive regions represent irreversible replacement fibrosis, where cardiomyocytes are completely replaced by collagen. These regions do not regress with medical therapy, so LGE is used to quantify established scar burden and predict long-term adverse events, rather than to monitor active reversible fibrotic processes.

To overcome the limitations of late gadolinium enhancement (LGE) and facilitate early detection of myocardial dysfunction, cardiac magnetic resonance functional tracking (CMR-FT) was developed. This technique analyzes cine images of the myocardium to assess its deformation during systole, allowing for the identification of subclinical dysfunction before ejection fraction abnormalities appear (149). Such dysfunction often correlates with diffuse fibrosis, serving as a biomarker that links myocardial microstructure to macroscopic function. Research indicates that in patients with hypertrophic cardiomyopathy, global longitudinal strain (GLS) and global circumferential strain (GCS) are compromised even when the left ventricular ejection fraction is normal. These impairments are associated with the extent of interstitial fibrosis, as shown by the extracellular volume fraction (ECV) (149, 150). In cases of dilated cardiomyopathy, combining myocardial strain parameters with ECV provides a comprehensive evaluation of the relationship between myocardial fibrosis and functional impairment. Thus, integrating LGE with feature tracking technology allows for a thorough and precise assessment of both focal and diffuse fibrosis and their functional impacts. CMR-FT-derived strain parameters reflect functional impairment caused by diffuse interstitial fibrosis and myocardial stiffness. Since early interstitial remodeling can improve with intervention, strain abnormalities may partially reverse after treatment, making functional imaging markers sensitive indicators of active, functionally relevant fibrotic processes.

Quantitative comparisons show that late gadolinium enhancement (LGE) is highly effective in detecting focal fibrosis. Conversely, combining CMR feature-tracking (CMR-FT) with extracellular volume (ECV) is particularly beneficial for assessing diffuse interstitial fibrosis and subclinical dysfunction. Mechanistically, LGE indicates irreversible collagen scar formation, while strain parameters and ECV reflect reversible interstitial remodeling. These methods collectively help differentiate between the two types of fibrosis, thereby supporting clinical decisions regarding anti-fibrotic therapies. Moreover, the use of supervised learning for intelligent identification of LGE lesion sites marks a significant technical advancement. Trained on expert-annotated focal fibrosis contours, this approach reduces the rate of missed diagnoses for small lesions.

## 7 Emerging multomics and integrated biomarker strategies

### 7.1 Non-coding RNAs (miRNAs, circRNAs)

Non-coding RNAs are crucial in regulating myocardial fibrosis, with microRNAs (miRNAs) like miR-21, miR-29, and miR-133 playing significant roles by targeting fibrosis-related signaling pathways. These miRNAs are consistently found in patients' peripheral blood, highlighting their potential as circulating biomarkers (151). For instance, elevated miR-21 levels in the plasma of obese patients are linked to cardiac fibrosis markers, primarily through the TGF-*β*1/Smad signaling pathway (151). Similarly, serum miR-133a levels are associated with reduced type I collagen carboxy-terminal propeptide (PICP) in patients after acute myocardial infarction (AMI), indicating its role in collagen metabolism regulation (93). Since 2022, evidence has shown that many circulating fibrotic miRNAs are encapsulated in extracellular vesicles, mainly exosomes, rather than existing freely (146). Exosomal miRNAs from cardiac fibroblasts and cardiomyocytes can cross cell membranes and function as paracrine signaling molecules, spreading fibrotic signals between cell types. Compared to cell-free circulating miRNAs, exosomal miRNAs are more resistant to RNase degradation and exhibit greater cardiac tissue specificity, making them more reliable for early non-invasive diagnosis (152). For example, exosomal miR-21 from pressure-overloaded cardiomyocytes can activate fibroblast proliferation and collagen production via the TGF-*β*/Smad pathway, with plasma levels correlating more closely with CMR-assessed ECV than total plasma miR-21 (153). This explains the inconsistency in free miRNA diagnostic performance across cohorts, as variations in extracellular vesicle enrichment affect total plasma miRNA quantification. In addition to miRNAs, circular RNAs (circRNAs) have emerged as promising biomarkers due to their high stability and tissue specificity, resulting from their closed circular structure. CircRNAs are resistant to degradation by exoribonucleases like RNase R due to the absence of free 5’ and 3’ ends (154). Many circulating circRNAs and miRNAs are encapsulated within exosomes and other extracellular vesicles, whose lipid bilayers protect nucleic acids from RNases in the bloodstream (155). Additionally, circulating non-coding RNAs often form stable ribonucleoprotein complexes with RNA-binding proteins such as Argonaute 2 (AGO2) and HuR, further preventing enzymatic degradation. Epitranscriptomic modifications, particularly N⁶-methyladenosine (m^6^A), can also enhance the stability of circRNAs by modulating their secondary structure and recruiting specific binding proteins (156). Similarly, circulating miRNAs rely on protective mechanisms like AGO2 protein binding, exosomal encapsulation, and association with high-density lipoproteins to maintain structural integrity in plasma, avoiding rapid renal clearance and nuclease degradation (157). However, this stability is not absolute; factors such as prolonged room temperature exposure, repeated freeze-thaw cycles, and severe hemolysis can degrade free circulating non-coding RNAs and damage extracellular vesicles, leading to 15%–30% deviations in quantification results and reduced inter-laboratory reproducibility. For example, circPAN3 is upregulated in the cardiac tissue of rats with myocardial infarction, where it binds to miR-221, regulating the FoxO3/ATG7 axis and contributing to autophagy-mediated cardiac fibrosis (158). These nucleic acid biomarkers offer high sensitivity and can enhance our understanding of the pathological mechanisms underlying fibrosis. Most circulating non-coding RNAs are not freely dissolved in plasma but are selectively packaged into extracellular vesicles, particularly exosomes, which protect them from RNase degradation and confer high stability in peripheral circulation. The packaging and release of exosomal non-coding RNAs are tightly regulated processes that reflect the activation state of parental cardiac fibroblasts.

Exosomes originate from the endosomal pathway, where early endosomes mature into multivesicular bodies (MVBs) through the inward budding of the limiting membrane, forming intraluminal vesicles (ILVs) (159). These ILVs are eventually released as exosomes. Two primary pathways facilitate the sorting of miRNAs into ILVs: the ESCRT-dependent pathway and the ESCRT-independent ceramide-dependent pathway. In activated cardiac fibroblasts, SUMOylated heterogeneous nuclear ribonucleoprotein A2B1 (hnRNPA2B1) and Y-box binding protein 1 (YBX1) serve as key RNA-binding proteins (160). They recognize specific sequence motifs in miRNA precursors and mediate their selective loading into exosomes. Under profibrotic conditions, such as TGF-*β*1 stimulation, the expression and post-translational modification of these sorting proteins change dynamically (161). This leads to distinct alterations in the exosomal miRNA cargo (162). For instance, activated cardiac fibroblasts selectively enrich miR-21 and decrease miR-29b in secreted exosomes, creating a circulating exosomal miRNA profile that accurately reflects the intracellular fibrogenic activation state.

**Figure 3:**
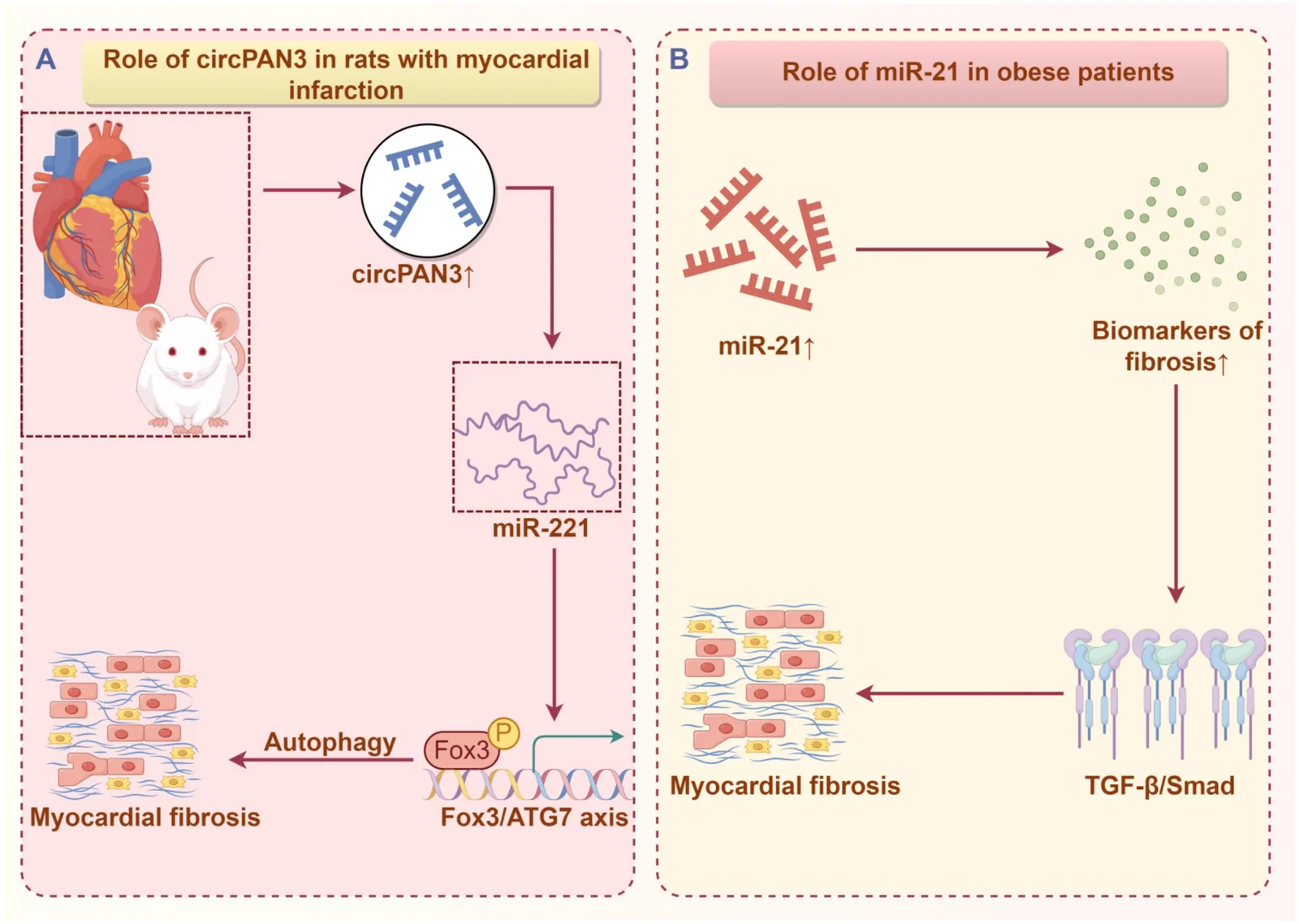
**(A)** In cardiac tissue from rat models of myocardial infarction, increased levels of circPAN3 influence the FoxO3/ATG7 signaling pathway by absorbing miR-221. This process contributes to myocardial fibrosis through autophagy. **(B)** In obese individuals, higher plasma miR-21 levels are linked to markers of myocardial fibrosis and facilitate fibrotic development by modulating the TGF-*β*1/Smad signaling pathway.

The release of exosomes is primarily regulated by members of the Rab GTPase family, notably Rab27a, Rab27b, and Rab11 (163). In conditions such as pressure overload or ischemic injury, profibrotic signaling pathways increase Rab27a expression in cardiac fibroblasts. This upregulation facilitates the docking and fusion of multivesicular bodies (MVBs) with the cell membrane, thereby enhancing exosome release into the cardiac interstitium and peripheral circulation (164). This controlled release ensures that the quantity and molecular composition of circulating exosomal miRNAs reflect the severity of ongoing myocardial fibrotic activation. These fibroblast-derived exosomal miRNAs function not only as biomarkers but also as paracrine mediators, transmitting fibrogenic signals to nearby cardiomyocytes and endothelial cells (25). This dual role establishes a mechanistic link between biomarker levels and disease progression. However, several challenges hinder the clinical application of these biomarkers. Key issues include the need to standardize detection methods, control costs, and identify the most effective diagnostic or prognostic combinations from the many candidate molecules available (165) (Figure 3).

Research on non-coding RNAs is revolutionizing the field, offering significant advancements beyond traditional markers. Unlike earlier animal-focused studies, extensive human cohort research has confirmed that plasma circPAN3, miR-21, and miR-133a exhibit high cardiac tissue specificity, providing 15%–20% greater diagnostic specificity than PICP/PIIINP. Notably, exosomal miRNA panels enhance diagnostic stability. A 2024 multicenter study reported that a 3-marker exosomal miRNA panel achieved an AUC of 0.89 for detecting diffuse myocardial fibrosis in hypertensive patients, with lower inter-individual variability than cell-free miRNA panels (166). Cardiac-enriched miRNA panels, featuring intrinsic tissue origin signatures, address the limitation of circulating biomarkers reflecting systemic rather than cardiac-specific processes. MicroRNAs such as miR-1, miR-133a, miR-208b, and miR-499 show highly restricted expression patterns, primarily in cardiomyocytes and cardiac fibroblasts. This tissue-restricted profile allows elevated levels in peripheral blood to be reliably traced to cardiac tissue, not extracardiac organs (167). Clinical validation studies indicate that a combined panel of these cardiac-enriched miRNAs achieves over 85% specificity in differentiating myocardial fibrosis from liver fibrosis and bone metabolic disorders, effectively addressing the poor organ specificity of traditional collagen metabolites. This approach is the most promising for capturing cardiac-specific fibrotic signals through minimally invasive peripheral blood testing (168). Mechanistic studies have revealed interactions between non-coding RNAs and core fibrotic pathways, such as TGF-*β* and autophagy, establishing a comprehensive “non-coding RNA-pathway-collagen metabolism” regulatory chain (169). This integrates multiple biomarker types mechanistically (170). Additionally, a panel combining five non-coding RNAs has been developed, achieving an AUC of 0.87 for early diagnosis of subclinical fibrosis, significantly outperforming traditional single circulating markers (171). However, challenges remain for clinical translation, primarily due to the lack of standardized detection methods and high testing costs (172). Non-coding RNAs act as upstream regulators of fibrogenic signaling, with expression changes occurring before histological scar formation. They predominantly reflect active, reversible cellular and molecular activation in early fibrosis, offering unique value for ultra-early detection of subclinical disease and prediction of fibrotic progression, rather than quantifying already formed scar tissue (173).

### 7.2 Proteomics and metabolomics findings

Advancements in high-throughput proteomics have revolutionized large-scale, unbiased screening, making it a powerful tool for discovering new circulating biomarkers of myocardial fibrosis (174). Unlike traditional single-molecule detection methods, proteomics offers a comprehensive profile of protein expression from peripheral blood, myocardium, and other samples. This holistic approach allows for the identification of differential proteins closely associated with the development of myocardial fibrosis (175). Recent high-quality cohort studies have identified several differentially expressed proteins, including plasminogen activator inhibitor-1 (PAI-1), insulin-like growth factor-binding protein 1 (IGFBP-1), and N-terminal pro-B-type natriuretic peptide (NT-proBNP) (174). These proteins strongly correlate with elevated extracellular volume fraction (ECV), the gold standard for assessing interstitial fibrosis. This finding highlights that myocardial interstitial fibrosis involves systemic protein expression changes detectable in peripheral blood (174). Beyond freely circulating proteins, many fibrosis-associated proteins are transported in peripheral circulation via exosomes (176). Their selective packaging is closely linked to the activation state of cardiac fibroblasts. During fibroblast activation, ESCRT complexes recognize ubiquitinated membrane and cytosolic proteins, sorting them into ILVs during MVB formation (177). Alternatively, proteins associated with lipid raft microdomains are incorporated into exosomes through ESCRT-independent mechanisms. Profibrotic matricellular proteins, such as periostin and thrombospondin-2, can be selectively packaged into fibroblast-derived exosomes. Their exosomal levels more accurately reflect local cardiac fibroblast activity than total serum concentrations, as exosomal cargo directly derives from the intracellular state of parental cells (178). The release of these protein-carrying exosomes follows the Rab GTPase-dependent secretory pathway, similar to non-coding RNAs, and their circulating concentrations increase with the intensity of interstitial fibrogenic remodeling (179).

Post-translational modifications (PTMs), particularly glycosylation and phosphorylation, significantly impact the stability, detectability, and functional activity of circulating fibrosis biomarkers. These modifications are a critical yet often overlooked source of variability in clinical assay performance (180). Glycosylation, for instance, affects profibrotic matricellular proteins like periostin and thrombospondin-2, which undergo extensive N-linked and O-linked glycosylation upon secretion from activated cardiac fibroblasts. Specific glycan structures protect these proteins from degradation by circulating proteases, extending their half-life in the bloodstream and altering their steady-state concentrations. Moreover, glycosylation can obscure or modify antibody-recognized epitopes on biomarker molecules (181). For example, variations in the sialylation of galectin-3 can change its oligomerization state and surface epitope structure, leading to inconsistent recognition by different commercial immunoassays and contributing to inter-laboratory result variability (182). In collagen-derived biomarkers such as PICP and PIIINP, the hydroxylation and glycosylation of pro-collagen peptides influence their molecular conformation and antigenicity. These factors must be considered during assay antibody development to ensure accurate quantification (183). Phosphorylation is another crucial PTM that affects biomarker function and detection. Proteins like thrombospondin-2 and periostin have multiple serine/threonine phosphorylation sites, and their phosphorylation status directly influences their binding affinity to integrins and extracellular matrix components, thereby modulating their paracrine profibrotic activity (13). Phosphorylated isoforms of matrix metalloproteinases exhibit altered enzymatic activity, affecting the generation rate of collagen degradation products and, consequently, the circulating levels of these turnover biomarkers. From an analytical standpoint, phosphorylation-induced conformational changes can decrease the binding affinity of detection antibodies to native proteins. This reduction can lead to underestimation of actual biomarker concentrations in standard sandwich immunoassays, compromising assay accuracy across various disease stages.

PAI-1 plays a vital role as an inhibitor in the fibrinolytic system. Its continuous elevation can hinder extracellular matrix degradation, accelerate collagen accumulation, and exacerbate myocardial fibrosis (184). Longitudinal studies indicate that combining PAI-1 detection with traditional collagen metabolites significantly improves the prediction of progressive myocardial fibrosis in patients with heart failure and hypertension. IGFBP-1 primarily regulates cell growth, repair, and inflammatory responses by modulating insulin-like growth factors' bioavailability (185). Abnormal IGFBP-1 expression can disrupt cardiomyocytes' and cardiac fibroblasts' self-repair processes post-injury, indirectly promoting fibrosis progression (174). Researchers have developed standardized intermediate phenotypic molecule (IMP) risk scoring models targeting anthracycline cardiotoxicity and hypertensive myocardial fibrosis (186). These models have undergone formal external validation in independent cohorts, marking a transition from exploratory omics screening to practical clinical risk assessment tools (187). The anthracycline-induced cardiotoxicity IMP score is a mechanism-driven proteomic-metabolomic model based on a phenotype-anchored selection strategy rather than unbiased high-throughput screening (188). Its core molecular panel includes four components mapping to distinct pathological intermediates of fibrogenesis: (1) PAI-1, indicating extracellular matrix degradation imbalance due to anthracycline-induced oxidative stress; (2) IGFBP7, marking cardiomyocyte senescence and paracrine activation of cardiac fibroblasts; (3) 9-hydroxyoctadecadienoic acid (9-HODE), a linoleic acid metabolite quantifying lipid peroxidation injury from anthracycline redox cycling; (4) ceramide d18:1/16:0, a bioactive lipid mediating cardiomyocyte apoptosis and subsequent fibrotic repair activation (189). This panel addresses three core pathological dimensions—oxidative damage, cellular senescence, and matrix remodeling—providing a comprehensive rationale for early subclinical fibrosis detection (190). External validation in a cohort of 327 breast cancer patients receiving doxorubicin chemotherapy yielded an AUC of 0.84, with 81.2% sensitivity and 78.6% specificity for detecting subclinical myocardial fibrosis (191). This model significantly outperforms classic single circulating markers: PIIINP achieves an AUC of 0.68 and Gal-3 an AUC of 0.71, representing a 13–16 percentage point improvement in diagnostic accuracy (192). For hypertensive myocardial fibrosis, the IMP-aligned multi-protein panel follows a similar pathway-stratified design, comprising PAI-1, IGFBP-1, and PIIINP (59). Each marker maps to an independent fibrogenic pathway, ensuring non-overlapping diagnostic information: PAI-1 reflects RAAS-mediated fibrinolytic system imbalance promoting extracellular matrix deposition; IGFBP-1 indicates dysregulated insulin-like growth factor signaling impairing cardiac tissue repair; PIIINP quantifies type III collagen synthesis rate. This complementary combination avoids redundant measurements and maximizes diagnostic value (193). External validation in a community-based cohort of 412 hypertensive patients achieved an AUC of 0.82, with 79.5% sensitivity and 76.3% specificity, and a net reclassification improvement (NRI) of 0.20 compared to PIIINP alone (194). From a biomarker discovery perspective, these IMP models offer an actionable, mechanism-anchored research paradigm distinct from traditional untargeted omics screening (195). Instead of selecting candidate molecules based solely on statistical fold change, researchers first define core intermediate pathological phenotypes of fibrosis (oxidative injury, fibroblast activation, matrix synthesis) and then match representative molecular markers for each phenotype. This phenotype-first, pathway-guided approach can be generalized to biomarker development for other etiological subtypes of myocardial fibrosis, reducing the high false-positive rate of unbiased omics screening and improving the translational success rate of candidate molecules. These validated performance metrics confirm that multi-omics models have advanced beyond preliminary screening and can be applied to clinical risk stratification of specific populations.

Metabolomics explores the dynamic changes in small-molecule metabolites within organisms, providing insights into metabolic phenotype alterations due to myocardial fibrosis at the terminal level of life activities (196). Myocardial fibrosis disrupts energy metabolism and collagen synthesis and cross-linking in myocardial tissue, creating a distinct “metabolic fingerprint” indicative of fibrotic activity. In doxorubicin-induced cardiotoxicity models, which illustrate drug-induced myocardial injury with fibrosis, multi-omics integration analysis has identified significant disruptions in the linoleic acid and arachidonic acid metabolic pathways (197). These lipid pathways are closely linked to inflammatory responses, with persistent inflammation significantly driving the progression of myocardial fibrosis (198). Additionally, long-term studies of patients with ST-segment elevation myocardial infarction (STEMI) show that serum levels of pro-III-collagen N-terminal peptide (PIIINP), a direct metabolite of collagen synthesis, consistently correlate with myocardial fibrosis severity as assessed by cardiac magnetic resonance imaging within one year post-onset (199). This evidence highlights the potential of characteristic metabolites as reliable metabolic biomarkers for quantitatively evaluating and monitoring myocardial fibrosis over the long term (200).

Proteomics and metabolomics provide significant advantages over traditional single biomarkers in studying fibrosis's molecular network, identifying early preclinical lesions, and distinguishing various etiological subtypes of fibrosis (201). However, this field faces notable challenges (202). The metabolite spectrum is highly influenced by factors such as diet, underlying metabolic diseases, and drug interventions, which can confound results (203). Additionally, the detection platforms for proteomics and metabolomics are expensive and involve complex experimental procedures (204). The absence of standardized detection protocols for clinical specimens further limits their widespread use in primary healthcare settings. For exosome-derived biomarkers, the lack of standardized isolation methods and techniques for tracing cell origin hampers their clinical application (205). Current assays struggle to definitively attribute circulating exosomal cargo to cardiac fibroblasts versus other cell types. Moreover, heterogeneous post-translational modifications of circulating biomarkers introduce additional analytical variability. Variations in glycosylation and phosphorylation across individuals and disease stages can alter antigen-antibody binding efficiency (206). This variability compromises assay precision and result comparability across different detection platforms, an underrecognized limitation of current clinical fibrosis biomarker assays.

### 7.3 Multi-biomarker integration models and artificial intelligence applications

The limited predictive power of individual biomarkers has driven the development of multivariate models incorporating multiple circulating biomarkers, imaging parameters, and clinical data (207). Integrating proteomic, metabolomic, and genomic data into unified predictive models presents two key challenges: the high dimensionality of multi-omics datasets, where molecular features far outnumber the sample size, and pervasive collinearity among interrelated molecular features within and across omics layers. To address these issues, specialized statistical and machine learning frameworks with built-in regularization and feature selection have become essential tools (208). For instance, combining thrombospondin-2, pro-III-collagen N-terminal peptide (PIIINP), and extracellular volume fraction (ECV) measured by cardiac MRI enhances the accuracy of predicting disease progression and adverse outcomes (209). A study showed that in patients with acute decompensated heart failure, using both PIIINP and NT-proBNP significantly improved the predictive model for all-cause mortality risk compared to NT-proBNP alone, as indicated by an increased area under the curve (AUC) (210). Moreover, artificial intelligence and machine learning algorithms can efficiently analyze high-dimensional multi-omics data and complex imaging features, uncovering intricate patterns beyond human analysis (211). Recent imaging AI research supports this advantage with quantitative performance data. A large-scale UK Biobank cohort study (*n* = 50,239 participants, 2025) developed supervised 2D U-Net deep learning segmentation models to automatically quantify native T1 mapping spatial features. The cohort was divided into training (*n* = 35,167), internal validation (*n* = 7,536), and held-out test (*n* = 7,536) sets, with training labels provided by two board-certified cardiovascular radiologists. The model achieved a Dice similarity coefficient of 0.84 for myocardial segmentation relative to expert manual contours and predicted long-term cardiovascular mortality with a C-index of 0.614, outperforming conventional single ECV measurement (C-index = 0.547). Another 2024 multicenter study developed a supervised 3D Vision Transformer (3D-ViT) non-contrast CMR AI model named MAARS for diffuse fibrosis screening. Trained on 8,213 patients from seven medical centers, the model was internally validated on 2,132 patients and externally tested on 2,127 independent patients from three additional centers. The reference standard was defined by three senior radiologists using ECV-confirmed diffuse fibrosis as the ground truth. Among 12,472 total enrolled heart failure patients, the model achieved an overall AUC of 0.89 (sensitivity 82.1%, specificity 80.7%), with a Cohen's kappa of 0.79 against expert consensus diagnosis, and improved to AUC = 0.93 for middle-aged high-risk subgroups, avoiding gadolinium contrast injection entirely. In multi-omics integrated machine learning pipelines, several externally validated predictive models have been reported, each employing targeted statistical strategies to manage high dimensionality and collinearity (212). A 2025 transcriptome-proteomics study applied least absolute shrinkage and selection operator (LASSO) regression and support vector machine (SVM) algorithms to identify 16 core fibrotic signature genes from 395 differential expression profiles (213). LASSO regression uses L1 regularization to shrink redundant feature coefficients to zero, achieving automatic feature selection and effectively addressing the high-dimensionality problem. The final multi-omics signature distinguished fibrotic heart failure patients from controls with AUC = 0.981 in internal cohorts and AUC = 0.895 in independent validation cohorts, with sensitivity 86.4% and specificity 90.2% (214). A 2026 proteomic UK Biobank study integrated 2,922 plasma protein markers and constructed an elastic-net logistic regression model for hypertrophic cardiomyopathy-related interstitial fibrosis. Elastic net combines L1 and L2 regularization terms, retaining LASSO's feature screening capability while stabilizing feature selection in highly correlated protein markers, making it suitable for handling collinearity in proteomic datasets (215). This model identified LTBP2 and ANGPT2 as core predictive proteins, with external replication AUC reaching 0.86 across two independent population cohorts (*n* > 90,000 total participants) (216). All regression pipelines incorporated baseline clinical risk factors as mandatory covariates to isolate the independent predictive contribution of proteomic signatures; after removing overlapping variance with age, blood pressure, LVEF, and renal function, the proteomic panel retained strong discriminatory capacity, confirming its unique prognostic value independent of conventional clinical indicators (217). Additionally, an XGBoost-based FibroScore multi-omics panel combining PIIINP, TIMP-1, and CMR-ECV was validated in three independent heart failure cohorts, yielding an overall AUC of 0.94 (95% CI: 0.91–0.97), far exceeding single collagen marker diagnostic efficiency (AUC 0.66–0.78) (218). Stratified analysis with clinical risk adjustment showed that FibroScore remained a significant independent predictor of cardiovascular death in subgroups with identical NYHA grade and LVEF, proving its predictive signal is not merely a surrogate for established clinical disease severity parameters. Multi-modal fusion machine learning models combining omics, serum biomarkers, and electronic medical records (EMR) also demonstrate robust clinical performance. A 2024 ensemble learning model integrating ECG features, circulating fibrosis markers, and clinical variables was validated on 14,052 patients; the CNN-based model achieved an AUC of 0.84 for detecting occult myocardial fibrosis, outperforming cardiologists' manual reading (AUC 0.63–0.66) (219). Methodologically, the widespread crosstalk and feedback loops among fibrogenic pathways mean that biomarker responses are inherently non-linear (220). Thus, simple linear weighted scoring panels have limitations in capturing complex fibrotic dynamics. Machine learning algorithms with non-linear fitting capabilities, such as XGBoost and neural networks, are better suited for integrating multi-class biomarkers, as they can implicitly model pathway synergistic and antagonistic interactions, improving the accuracy of dynamic fibrosis progression prediction. Despite the promising performance of these integrated models, the current literature generally lacks a standardized, principled framework for selecting, weighting, and combining biomarkers across different classes (221). Optimizing diagnostic yield while minimizing biomarker redundancy and testing costs requires systematic panel design rules rather than empirical indicator stacking (222). A four-principled framework for multi-biomarker panel construction is proposed as follows:

1. Hierarchical biomarker selection strategy based on pathophysiological complementarity

Biomarkers are categorized into three tiers to ensure comprehensive coverage of independent fibrotic dimensions while avoiding redundancy (140). The core tier includes well-validated, cost-effective markers that represent distinct pathophysiological axes: one for active ECM turnover (e.g., PIIINP), another for cardiac-specific matrix remodeling (e.g., TSP-2), and a third for structural fibrotic burden when imaging is available (e.g., ECV) (223). These markers are chosen from different signaling pathways to reduce collinearity, forming the minimal effective panel. The supplementary tier consists of phenotype-specific markers added based on clinical context (224). For instance, Gal-3 is included for patients with inflammatory-predominant HFpEF, while MMP/TIMP ratios are used for those with suspected active matrix degradation imbalance. The exploratory tier features novel non-coding RNAs and omics markers. These are reserved for high-risk subclinical populations or research settings, offering ultra-early molecular signals but are not recommended for routine clinical use (225).

2. Standardized weighting assignment paradigms

Three complementary weighting approaches can be applied to cardiac fibrosis biomarker panels, balancing statistical performance, mechanistic rationality, and clinical feasibility (226). The first approach is statistics-driven weighting. Here, regression coefficients from LASSO, elastic-net, or multivariable Cox models in large, representative cohorts are used to assign continuous weights based on each marker's independent diagnostic or prognostic contribution, as seen in the FibroScore model. This method optimizes statistical performance but requires multicenter training datasets to prevent overfitting (227). The second approach is mechanism-driven grading weighting (228). In this method, markers are assigned equal or graded weights depending on the independence of their underlying pathophysiological pathways. This ensures that each core fibrogenic axis—such as inflammatory activation, fibroblast proliferation, collagen synthesis, matrix cross-linking, and structural scarring—is appropriately represented. The third approach is clinical utility-adjusted weighting. Here, weights are moderately adjusted based on testing cost, turnaround time, and accessibility. This adjustment enhances the practicality of the panels, especially in primary care or resource-limited settings.

3. Explicit redundancy reduction rules

To avoid unnecessary panel expansion without significant performance improvement, explicit control of collinearity is essential during panel development (229). Markers that indicate the same fibrotic phase or are part of the same downstream signaling pathway should not be included together in standard panels (230). For example, PICP and ICTP both indicate type I collagen metabolism and exhibit high covariance; therefore, only one should be included in basic panels unless detailed collagen turnover staging is necessary. Similarly, including multiple downstream effector molecules of the TGF-*β* pathway is discouraged, as they offer limited additional diagnostic value due to shared variance (231). Variance inflation factor (VIF) testing and pairwise correlation analysis are recommended as quantitative methods to identify and eliminate redundant indicators (232).

4. Graded cost-performance matching for different clinical scenarios

To optimize diagnostic accuracy relative to medical resource allocation, a tiered panel configuration strategy is recommended. For population-level screening and primary care follow-up, a cost-effective approach involves using a minimal panel of 2–3 circulating markers, such as PIIINP, TSP-2, and NT-proBNP (233). This strategy maximizes cost-effectiveness. In contrast, high-risk patients who need precise fibrosis staging and prognostic stratification benefit from a combined panel that includes circulating biomarkers and CMR structural indicators. This approach balances diagnostic accuracy with acceptable costs (234). For ultra-early detection in research cohorts or precision medicine contexts, multi-omics and non-coding RNA panels are suitable. However, their high cost limits their routine clinical use (235). This graded configuration ensures that the diagnostic yield aligns with testing expenditures.

## 8 Challenges, outlook, and future directions

### 8.1 Current challenges facing biomarkers

The clinical application of biomarkers for myocardial fibrosis faces significant challenges. Primarily, the lack of cardiac tissue specificity is a major limitation of circulating biomarkers. The concentration of a biomarker in peripheral blood reflects its release from all organs and tissues, not just the heart. For widely used markers like PICP, PIIINP, and Gal-3, which are expressed in various organs, levels can increase due to fibrosis in the liver, kidneys, and bones. This reduces their specificity for diagnosing myocardial fibrosis, as they are susceptible to non-cardiac diseases. Importantly, traditional circulating biomarkers lack a tissue origin signature, preventing clinicians from distinguishing whether elevated levels are due to cardiac fibrotic remodeling or other pathological processes (236). This deficiency leads to false-positive diagnoses and inaccurate fibrosis quantification in patients with multiple organ comorbidities. A critical, often overlooked issue exacerbates this limitation: most published validation studies for myocardial fibrosis biomarkers exclude patients with extracardiac fibrotic diseases, such as liver cirrhosis, advanced chronic kidney disease, and interstitial pulmonary fibrosis (174). This selection bias means that diagnostic performance metrics like AUC, sensitivity, and specificity are based on populations with isolated cardiac fibrosis and cannot be directly applied to patients with multi-organ fibrotic conditions (237). In real-world clinical settings, where elderly heart failure patients frequently exhibit combined hepatic, renal, and pulmonary fibrotic changes, the diagnostic specificity of traditional circulating markers declines sharply. Moreover, their ability to quantify the cardiac-specific contribution to systemic fibrotic burden remains largely unvalidated.

Emerging strategies to address cross-organ source interference in cardiac diagnostics can be categorized into three main approaches. First, cardiac-enriched non-coding RNA panels utilize the specific expression patterns of molecules like miR-1, miR-133a, and miR-208b to differentiate cardiac signals from extracardiac noise (238). Second, cardiac cell-targeted extracellular vesicle subtyping isolates exosome subpopulations with cardiomyocyte- or cardiac fibroblast-specific markers, allowing for precise measurement of cardiac-specific molecular release without interference from other fibrotic organs (239). Third, tissue-specific post-translationally modified collagen peptides identify unique collagen cross-linking or hydroxylation patterns specific to cardiac tissue, potentially providing organ specificity even for traditional collagen turnover markers (240). Despite their promise, these methods are largely in early exploratory stages or small-sample validations, and their diagnostic accuracy in patients with concurrent multi-organ fibrosis has not been systematically assessed (241). Myocardial fibrosis is a dynamic and complex process that involves a balance between collagen synthesis, deposition, and degradation (242). Biomarkers measured at a single time point, such as PICP or PIIINP, cannot capture these dynamic changes or indicate whether fibrosis is progressing or regressing, limiting their utility in assessing disease progression (242). Moreover, extensive crosstalk and feedback loops among fibrogenic signaling pathways result in non-linear biomarker responses. Circulating biomarker levels do not maintain a simple linear relationship with the histological severity of fibrosis. Positive feedback loops may cause rapid biomarker escalation once fibrogenesis exceeds a certain threshold, while negative regulatory mechanisms may stabilize biomarker levels during chronic disease. This non-linearity complicates the inference of disease progression from single or intermittent biomarker measurements, and linear extrapolation may lead to inaccurate clinical judgments. Myocardial fibrosis also exhibits significant etiological heterogeneity. Fibrosis from different causes, such as post-myocardial infarction replacement fibrosis versus diffuse fibrosis from hypertension or diabetes, may have distinct molecular and pathological characteristics (242). Most existing biomarkers cannot differentiate between these fibrosis phenotypes, falling short of precision medicine needs (243). Additionally, pre-analytical variability from sample processing and storage conditions introduces measurement bias, compromising biomarker comparability across studies and clinical centers (244). For protein biomarkers like PICP, PIIINP, and Gal-3, prolonged room temperature incubation after blood collection can lead to degradation by proteases, causing 10%–25% measurement deviation within 6 h. Repeated freeze-thaw cycles further disrupt protein structure and reduce detectable concentrations, with collagen-derived peptide markers particularly susceptible (245). Circulating miRNA biomarkers, despite their stability from protein binding and exosomal encapsulation, degrade significantly after more than three freeze-thaw cycles. Hemolysis during sample collection can release intracellular miRNAs and proteases, contaminating biomarker measurements and generating false positives (246). The correlation between circulating biomarkers and invasive histopathological fibrosis quantification, the diagnostic gold standard, lacks consistent validation (247). Endomyocardial biopsy studies show only moderate concordance between circulating markers and myocardial collagen volume fraction, with correlation coefficients generally ranging from 0.3 to 0.6. This discrepancy stems from three main sources: (1) Sampling bias from endomyocardial biopsy, which captures only a small fraction of myocardial tissue and may not reflect the overall burden of left ventricular fibrosis (248); (2) Biological non-specificity, as most circulating collagen metabolites reflect systemic extracellular matrix turnover, confounded by concurrent fibrosis in other organs; (3) Methodological heterogeneity, with no universal standard for histological fibrosis quantification, leading to poor comparability of results across studies (249). For example, one study found a significant correlation between left ventricular extracellular volume fraction (ECV) and levels of C-terminal cross-linked type I collagen (ICTP) in the coronary sinus, but associations with other markers were inconsistent (250). This inconsistency undermines the reliability of biomarkers for clinical decision-making, limiting their role to adjunctive assessment tools rather than independent diagnostic criteria (251). Additionally, early biomarker research often failed to adjust for established clinical risk factors in prognostic analyses. Many single-cohort studies reported unadjusted survival associations between biomarkers and adverse outcomes, making it unclear whether the predictive effect was due to intrinsic fibrotic signals or confounding clinical variables (e.g., advanced age, impaired renal function, severe ventricular dysfunction, uncontrolled hypertension) (252). Without multivariate adjustment, it is impossible to determine if biomarkers provide unique prognostic information beyond conventional clinical risk stratification systems (207). This gap has been addressed in post-2022 multicenter prospective trials, which uniformly use fully adjusted Cox regression models to quantify independent hazard ratios and incremental predictive gains of biomarkers.

Integrated biomarker models, multi-omics platforms, and AI applications encounter significant practical challenges that current research has yet to fully resolve (253). First, there is a lack of evidence and insufficient external validation for combined biomarker panels and AI algorithms (254). Most models are developed and validated using single-center retrospective cohorts, lacking the large-scale, multicenter, prospective external validation necessary for broader applicability. Variability in patient demographics, underlying diseases, detection platforms, and operating protocols across different medical centers often diminishes model accuracy and generalizability when applied to external populations (255). Second, high costs and limited accessibility impede clinical adoption. High-throughput proteomics, metabolomics, and next-generation sequencing for non-coding RNAs require advanced experimental platforms, skilled technicians, and costly reagents, making them unaffordable for primary medical institutions and routine clinical screening (256). Additionally, AI-assisted cardiac image analysis relies on high-quality CMR data and customized algorithm training, further increasing medical costs and limiting its use in resource-limited regions (257).

### 8.2 Future research directions

Research on myocardial fibrosis biomarkers is advancing through integration and innovation to address existing challenges. A multi-biomarker strategy is crucial for enhancing diagnostic accuracy and prognostic predictions, as single biomarkers often fail to capture the complex pathophysiology of fibrosis. Integrating biomarkers from various pathways into multivariate models offers superior predictive value (251). For instance, combining brain natriuretic peptide (BNP), galectin-3 (Gal-3), PIIINP, and thrombospondin-2 improves predictions for left ventricular hypertrophy and diastolic dysfunction beyond clinical variables alone (251). When developing combined panels, researchers should adhere to a principled design framework that includes hierarchical selection, standardized weighting, and redundancy control, rather than merely accumulating biomarkers (258). Emphasis should be placed on large-sample, multicenter prospective validation to ensure model stability and generalizability. Cost-effectiveness comparisons of different panel combinations should be conducted to identify optimal configurations for specific clinical scenarios. Future panels should integrate two types of biomarkers: one reflecting irreversible scar burden for disease staging and prognosis, and another capturing active fibrogenic activity for early detection and treatment monitoring (140). This dual-axis evaluation system will enable clinicians to differentiate between reversible active disease and irreversible established scar, guiding personalized intervention decisions (259). Future research should also explore cell-type-specific exosomal biomarkers based on their selective packaging mechanisms to enhance the cardiac specificity of circulating fibrosis biomarkers and enable non-invasive tracing of cellular-level fibrotic activation (260). In terms of analytical methodology, future assay development should consider post-translational modification profiles and develop modification-specific detection antibodies or mass spectrometry-based targeted assays to improve the accuracy and consistency of biomarker quantification across different platforms and populations. Additionally, stratified cut-off values should be established for different age groups, renal function statuses, and underlying diseases to minimize diagnostic bias from confounding factors. All future prognostic validation workflows must prioritize multivariate adjustment for standardized clinical risk factors to rigorously quantify the independent prognostic contribution of each biomarker panel, calculate incremental C-index/AUC compared with pure clinical prediction models, and clearly report whether biomarker signals remain statistically significant after controlling for conventional disease severity parameters (143). Exploring biomarkers' roles in dynamic monitoring and treatment response assessment is also crucial. With the advent of novel anti-fibrotic drugs like ARNIs, SGLT2i, and MRAs, reliable biomarkers with clear temporal correlation to functional recovery are urgently needed as surrogate endpoints in clinical trials. Current evidence outlines a general temporal hierarchy of biomarker responses: circulating collagen synthesis markers such as PIIINP show the earliest changes (significant reduction at 8–12 weeks after treatment initiation), followed by MMP/TIMP ratio shifts at 3–6 months, then CMR-derived native T1 and ECV improvements at 6–12 months, and ultimately overt improvements in cardiac ejection fraction and diastolic function at 12–24 months (261). Studies confirm that sacubitril/valsartan significantly reduces TIMP-1 levels in HFpEF patients as early as 12 weeks, and the magnitude of TIMP-1 reduction predicts subsequent improvement in exercise capacity at 6 months. However, the quantitative dose-response relationship between biomarker change amplitude and functional improvement degree remains incompletely established, and there is still a lack of unified threshold values for defining “biological response” to anti-fibrotic therapy across different drug classes. Future prospective studies with serial multi-timepoint sampling are required to further validate the temporal causal relationship between biomarker trajectories and long-term clinical outcomes (262). In terms of novel biomarker discovery, future research should shift from non-specific systemic collagen metabolites to pathway-specific molecular markers grounded in validated fibrogenic signaling cascades. Priority should be given to identifying pathway-specific cleavage products (such as LTBP-1 fragments for TGF-*β* pathway activation and soluble IL-11R*α* for inflammatory fibrotic signaling) and post-translationally modified collagen neo-epitopes (263). These can overcome the organ non-specificity of traditional collagen metabolites and substantially improve diagnostic specificity for cardiac fibrosis. These pathway-specific markers can not only quantify fibrotic burden but also indicate the dominant pathogenic signaling axis in individual patients, laying the foundation for precision anti-fibrotic therapy selection (209).

Integrating multi-omics approaches with artificial intelligence holds great potential for uncovering specific biomarker combinations and novel pathophysiological mechanisms. By combining proteomics, metabolomics, and single-cell sequencing with machine learning algorithms, researchers can explore the molecular networks underlying myocardial fibrosis, leading to the discovery of new biomarkers and therapeutic targets. For example, multi-omics analysis has identified distinct molecular subtypes in patients with dilated cardiomyopathy (DCM), resulting in the discovery of novel prognostic biomarkers such as interleukin-4 receptor-alpha (IL-4R*α*). From a methodological standpoint, future clinical translation necessitates the standardization of statistical analytical pipelines for high-dimensional multi-omics data (264). This includes establishing unified preprocessing specifications, criteria for selecting regularization parameters, and workflows for diagnosing collinearity to ensure the reproducibility and generalizability of integrated models across various centers. However, it is crucial to acknowledge that multi-omics and AI technologies are still in the early stages of translation. Before they can be widely implemented in clinical settings, researchers must tackle several challenges: standardizing experimental and data analysis protocols, streamlining detection workflows to control medical costs, and refining AI algorithms to improve model interpretability.

Integrating non-invasive imaging with liquid biopsy is essential for accurately classifying fibrosis phenotypes. By combining imaging biomarkers such as CMR-ECV and T1 mapping with liquid biopsy techniques—including circulating biomarkers, extracellular vesicles, and circulating cell-free DNA—this approach leverages their complementary strengths to provide comprehensive insights into fibrosis (242). This strategy enables earlier and more precise identification of high-risk patients, guides personalized treatment, and ultimately improves patient outcomes (242). Evaluating cost-effectiveness is crucial when implementing this integrated approach. For asymptomatic low-risk populations, conventional serum biomarkers remain the preferred screening tools due to their affordability and ease of use. Conversely, the combination of imaging and liquid biopsy should be reserved for confirmed high-risk patients to avoid unnecessary medical examinations and financial burdens. Additionally, long-term follow-up cohorts are needed to assess the clinical value of these integrated strategies in reducing adverse cardiovascular events and enhancing long-term prognosis.

## 9 Conclusion

Myocardial fibrosis is a key pathological mechanism in the progression of various cardiovascular diseases and is linked to poor prognosis. Thus, accurately assessing this condition is clinically crucial. Unlike previous reviews that mainly offered descriptive summaries of classic biomarkers, this article highlights research achievements since 2022. It systematically differentiates between incremental updates and original innovations in the field. Traditional collagen metabolites like PICP and PIIINP, along with Gal-3, thrombospondin-2, and other circulating protein markers, represent mostly incremental progress. However, these markers have limitations, such as low specificity and inconsistent efficacy across different populations. Since 2022, CMR imaging biomarkers have seen technical advancements and AI optimization, reflecting both technology upgrades and model innovations. In contrast, non-coding RNA, multi-omics biomarkers, and AI-integrated models represent the most significant original breakthroughs in recent years. These offer early diagnostic potential and high tissue specificity. Additionally, we emphasize that post-translational modifications, such as glycosylation and phosphorylation, are crucial for biomarker stability, assay performance, and functional activity. These factors should be integrated into future assay optimization and clinical result interpretation. Furthermore, we stress that fibrogenic signaling pathways form a complex regulatory network with extensive crosstalk and feedback loops, leading to inherently non-linear dynamic responses of circulating biomarkers. This network property must be fully considered in clinical interpretation and multi-biomarker model construction, rather than relying on a simple linear superposition perspective.

Biomarkers across various dimensions offer distinct advantages and roles in medical practice. Circulating biomarkers, such as thrombospondin-2 (TSP-2), Galectin-3 (Gal-3), and PIIINP, are particularly beneficial due to their ease of collection and capacity for dynamic monitoring. They effectively indicate systemic or localized inflammatory and fibrotic activity, making them valuable tools for outpatient screening and long-term follow-up. After adjusting for established clinical risk factors like age, renal function, NYHA classification, LVEF, and chronic comorbidities, TSP-2, Gal-3, and cardiac magnetic resonance extracellular volume fraction (CMR ECV) maintain independent prognostic predictive power. They provide additional risk stratification information not available from conventional clinical parameters. Conversely, traditional collagen metabolites such as PIIINP and PICP lose independent predictive value in elderly populations with multiple comorbidities, as their prognostic signals largely overlap with routine clinical severity indicators. The extracellular volume fraction (ECV) measured by cardiac magnetic resonance (CMR) is considered the “gold standard” for non-invasive myocardial tissue assessment. It accurately quantifies diffuse fibrosis and offers detailed structural information. These two approaches—circulating biomarkers and imaging—are complementary, forming a framework of “functional activity” and “structural changes.” In clinical practice, balancing these methods involves using the dynamic sensitivity of circulating biomarkers for risk prediction and initial treatment efficacy assessment. This is followed by precise quantification through ECV in cardiac MRI for diagnosis and baseline evaluation. This strategy facilitates a seamless transition from screening to diagnosis and from qualitative to quantitative assessment.

Research into novel molecular biomarkers, such as non-coding RNA and multi-omics, is a promising field. These biomarkers illuminate the mechanisms behind fibrosis onset and progression by analyzing gene expression regulation and molecular network interactions. They offer insights into identifying new therapeutic targets. Notably, these molecular changes may emerge earlier than traditional biomarkers, highlighting their potential for “ultra-early” diagnosis. However, we must realistically evaluate their clinical applicability. Currently, high detection costs, lack of standardized protocols, and insufficient clinical validation hinder their routine use. Most studies remain in exploratory and single-center validation phases, making it imprudent to overestimate their immediate clinical value. Rigorous, large-scale, multicenter, prospective cohort studies are urgently needed to confirm the independent predictive value of these biomarkers, establish optimal cutoff values, and assess the benefits of integrating them into existing diagnostic systems.

The future of myocardial fibrosis biomarker development focuses on standardization, integration, and personalization. Standardizing the testing and interpretation of various biomarkers, including novel circulating molecules and imaging parameters, is essential for ensuring research comparability and clinical applicability. Progress will not depend on discovering a single “universal biomarker.” Instead, it will emerge from using advanced algorithms, like artificial intelligence, to integrate circulating biomarkers, imaging features, clinical parameters, and genomic data into multidimensional “integrated biomarker panels.” These models will provide a comprehensive assessment of fibrosis activity, extent, etiology, and potential reversibility, enabling precise risk stratification beyond what single indicators can achieve. Ultimately, this system will transform clinical practice by allowing physicians to identify high-risk patients before irreversible myocardial remodeling occurs, facilitating early, targeted interventions tailored to specific fibrotic drivers. This approach will promote proactive, individualized management of myocardial fibrosis, significantly improving the long-term prognosis for patients with heart failure and cardiomyopathy.

## References

[1] Scuruchi M Mannino F Imbesi C Pallio G Vermiglio G Bagnato G. Biglycan involvement in heart fibrosis: modulation of adenosine 2A receptor improves damage in immortalized cardiac fibroblasts. Int J Mol Sci. (2023) 24(2):1784. 10.3390/ijms24021784 36675295 PMC9866951

[2] Niro F Fernandes S Cassani M Apostolico M Oliver-De La Cruz J Pereira-Sousa D. Fibrotic extracellular matrix impacts cardiomyocyte phenotype and function in an iPSC-derived isogenic model of cardiac fibrosis. Transl Res. (2024) 273:58–77. 10.1016/j.trsl.2024.07.003 39025226 PMC11832458

[3] Singh R Kaundal RK Zhao B Bouchareb R Lebeche D. Resistin induces cardiac fibroblast-myofibroblast differentiation through JAK/STAT3 and JNK/c-Jun signaling. Pharmacol Res. (2021) 167:105414. 10.1016/j.phrs.2020.105414 33524540 PMC8085100

[4] Al Akhali ES Alshoabi SA Hamid AM Alsultan KD Omer AM Alhammadi MA. Cardiac amyloidosis: a diagnostic challenge. Radiol Case Rep. (2024) 19(11):4730–5. 10.1016/j.radcr.2024.07.119 39228955 PMC11366889

[5] Paw M Kusiak AA Nit K Litewka JJ Piejko M Wnuk D. Hypoxia enhances anti-fibrotic properties of extracellular vesicles derived from hiPSCs via the miR302b-3p/TGFβ/SMAD2 axis. BMC Med. (2023) 21(1):412. 10.1186/s12916-023-03117-w 37904135 PMC10617123

[6] Zou Z Han J Zhu Z Zheng S Xu X Liu S. Transforming heart transplantation care with multi-omics insights. J Transl Med. (2025) 23(1):710. 10.1186/s12967-025-06772-0 40598485 PMC12211886

[7] Pei J Zhao X Pang Y Xu S Li C Wang S. FAP In cardiovascular pathophysiology: advances in non-invasive imaging and therapeutic targeting for myocardial fibrosis. Semin Nucl Med. (2026) 56(4):719–35. 10.1053/j.semnuclmed.2026.02.007 41916840

[8] Screever EM Gorter TM Willems TP Aboumsallem JP Suthahar N Mahmoud B. Diffuse myocardial fibrosis on cardiac magnetic resonance imaging is related to galectin-3 and predicts outcome in heart failure. Biomolecules. (2023) 13(3):410. 10.3390/biom13030410 36979345 PMC10046101

[9] Hemmati MA Monemi M Asli S Mohammadi S Foroozanmehr B Haghmorad D. Using new technologies to analyze gut microbiota and predict cancer risk. Cells. (2024) 13(23):1987. 10.3390/cells13231987 39682735 PMC11640725

[10] Tsai CR Martin JF. Hippo signaling in cardiac fibroblasts during development, tissue repair, and fibrosis. Curr Top Dev Biol. (2022) 149:91–121. 10.1016/bs.ctdb.2022.02.010 35606063 PMC10898347

[11] Braga YLL do Carmo Neto JR Franco PIR Helmo FR Dos Reis MA de Oliveira FA. The influence of IL-11 on cardiac fibrosis in experimental models: a systematic review. J Cardiovasc Dev Dis. (2024) 11(2):65. 10.3390/jcdd11020065 38392279 PMC10888948

[12] Meng K Cai H Cai S Hong Y Zhang X. Adiponectin modified BMSCs alleviate heart fibrosis via inhibition TGF-beta1/smad in diabetic rats. Front Cell Dev Biol. (2021) 9:644160. 10.3389/fcell.2021.644160 33829019 PMC8019808

[13] Zhang N Wu X Zhang W Sun Y Yan X Xu A. Targeting thrombospondin-2 retards liver fibrosis by inhibiting TLR4-FAK/TGF-β signaling. JHEP Rep Innov Hepatol. (2024) 6(3):101014. 10.1016/j.jhepr.2024.101014 PMC10877131 38379585

[14] Kimura T Iwadare T Wakabayashi SI Kuldeep S Nakajima T Yamazaki T. Thrombospondin 2 is a key determinant of fibrogenesis in non-alcoholic fatty liver disease. Liver Int. (2024) 44(2):483–96. 10.1111/liv.15792 38010940

[15] Miftode RS Petriș AO Onofrei Aursulesei V Cianga C Costache II Mitu O. The novel perspectives opened by ST2 in the pandemic: a review of its role in the diagnosis and prognosis of patients with heart failure and COVID-19. Diagnostics (Basel). (2021) 11(2):175. 10.3390/diagnostics11020175 33530550 PMC7911622

[16] Wang X Han SJ Wang XL Xu YF Wang HC Peng JY. Soluble ST2 is a biomarker associated with left ventricular hypertrophy and concentric hypertrophy in patients with essential hypertension. Am J Hypertens. (2024) 37(12):987–94. 10.1093/ajh/hpae105 39136164 PMC11565189

[17] Perez LM Venugopal SV Martin AS Freedland SJ Di Vizio D Freeman MR. Mechanisms governing lineage plasticity and metabolic reprogramming in cancer. Trends Cancer. (2024) 10(11):1009–22. 10.1016/j.trecan.2024.08.001 39218770

[18] Qin X Guo W Wang Y Cai X Xiao J. A pathological collagen-targeting nanozyme chip for ultrasensitive quantification of collagen in body fluids. Anal Chem. (2025) 97(42):23299–311. 10.1021/acs.analchem.5c04014 41081707

[19] Guo Y Lin L Xu K Zhao S Sun G Chen Y. Myocardial native T1 and extracellular volume measurements at 5T: feasibility study and initial experience. J Cardiovasc Magn Reson. (2025) 27(1):101896. 10.1016/j.jocmr.2025.101896 40268169 PMC12182820

[20] Du J Zhou M Wang H Wang J Raffield LM Zhou R. Multi-omics integration predicts the incidence of 17 diseases in the UK biobank. Kidney Int. (2002) 62:237–44. 10.1038/s41467-026-73017-z 42106324 PMC13376913

[21] Chistyakov DV Nikolskaya AI Gorbatenko VO Goriainov SV Prishchep PL Kuzmin YB. Multi-omics profiling of oxylipins and gene expression in pediatric sarcomas identifies metastatic signatures and prognostic models. Free Radic Biol Med. (2026) 253:195–207. 10.1016/j.freeradbiomed.2026.05.310 42177999

[22] Liang Y Xu Y Ding L Chen X Li H. Urotensin II induces cardiac fibrosis through the TGF-β/Smad signaling pathway during the development of cardiac hypertrophy. Int Heart J. (2021) 62(5):1135–44. 10.1536/ihj.21-032 34588407

[23] Wu H Jiang W Pang P Si W Kong X Zhang X. m(6)A reader YTHDF1 promotes cardiac fibrosis by enhancing AXL translation. Front Med. (2024) 18(3):499–515. 10.1007/s11684-023-1052-4 38806989

[24] Lai Q Liu FM Rao WL Yuan GY Fan ZY Zhang L. Aminoacylase-1 plays a key role in myocardial fibrosis and the therapeutic effects of 20(S)-ginsenoside Rg3 in mouse heart failure. Acta Pharmacol Sin. (2022) 43(8):2003–15. 10.1038/s41401-021-00830-1 34916608 PMC9343399

[25] Prieto-Vila M Yoshioka Y Kuriyama N Okamura A Yamamoto Y Muranaka A. Adult cardiomyocytes-derived EVs for the treatment of cardiac fibrosis. J Extracell Vesicles. (2024) 13(7):e12461. 10.1002/jev2.12461 38940266 PMC11211925

[26] Liu S Wang Q Min J Zhang Z Zhang Y Yang J. The CCL20-integrin α5β1 interaction enhances TGF-β/Smad signaling to promote fibroblast activation in pulmonary fibrosis. Nat Commun. (2025) 16(1):9183. 10.1038/s41467-025-64211-6 41102188 PMC12532996

[27] Liu CH Lee HS Liou JP Hua HS Cheng WH Yuliani FS. MPT0E028, a novel pan-HDAC inhibitor, prevents pulmonary fibrosis through inhibition of TGF-β-induced CTGF expression in human lung fibroblasts: involvement of MKP-1 activation. Eur J Pharmacol. (2024) 977:176711. 10.1016/j.ejphar.2024.176711 38839029

[28] Livingston MJ Zhang M Kwon SH Chen JK Li H Manicassamy S. Autophagy activates EGR1 via MAPK/ERK to induce FGF2 in renal tubular cells for fibroblast activation and fibrosis during maladaptive kidney repair. Autophagy. (2024) 20(5):1032–53. 10.1080/15548627.2023.2281156 37978868 PMC11135847

[29] Roger I Montero P Milara J Cortijo J. Pirfenidone and nintedanib attenuates pulmonary artery endothelial and smooth muscle cells transformations induced by IL-11. Eur J Pharmacol. (2024) 972:176547. 10.1016/j.ejphar.2024.176547 38561103

[30] Hu Y Jiang H Xu Y Chen G Fan R Zhou Y. Stomatin-like protein 2 deficiency exacerbates adverse cardiac remodeling. Cell Death Discov. (2023) 9(1):63. 10.1038/s41420-023-01350-z 36788223 PMC9929064

[31] Kalailingam P Rannikmae K Hausman-Kedem M Musolino PL Ruigrok YM. Genetic insights into hemorrhagic stroke and vascular malformations: pathogenesis and emerging therapeutic strategies. Stroke. (2025) 56(5):1298–311. 10.1161/strokeaha.124.045182 40084704 PMC12037314

[32] Jopek K Tyczewska M Szyszka M Blatkiewicz M Jopek M Malendowicz LK. Impact of classic adrenal secretagogues on mRNA levels of urotensin II and its receptor in adrenal gland of rats. Int J Mol Sci. (2023) 24(17):13412. 10.3390/ijms241713412 37686217 PMC10488159

[33] Shi Z Wen K Fu W Zou Z Guo K Sammudin NH. mRNA m(6)A modifications and the RNA-binding protein YTHDF1 affect translational control in both normal and pathological learning. Proc Natl Acad Sci U S A. (2026) 123(16):e2518250123. 10.1073/pnas.2518250123 41973927 PMC13099619

[34] Zhang L Zhang Y Liu J Li H Liu B Zhao T. N6-methyladenosine mRNA methylation is important for the light response in soybean. Front Plant Sci. (2023) 14:1153840. 10.3389/fpls.2023.1153840 37082338 PMC10110966

[35] Zhang F Zeng L Zou K Lin K Yao F Song J. Lactylation-driven YTHDC1 alleviates MASLD by suppressing PTPN22-mediated dephosphorylation of NLRP3. Adv Sci (Weinh). (2026) 13(20):e10192. 10.1002/advs.202510192 41298230 PMC13067837

[36] Tian L Wang Y Li LX Agborbesong E Zhou JX Mou S. Alanyl-tRNA synthetase 1 and cyst growth in autosomal dominant polycystic kidney disease. J Am Soc Nephrol. (2023) 34:1823–42. 10.1681/asn.0000001169 42329029

[37] Wood CS Pennel KAF Leslie H Legrini A Cameron AJ Melissourgou-Syka L. Spatially resolved transcriptomics deconvolutes prognostic histological subgroups in patients with colorectal cancer and synchronous liver metastases. Cancer Res. (2023) 83(8):1329–44. 10.1158/0008-5472.Can-22-2794 37057593 PMC10102851

[38] Mo J Huang H Zhu B Liao R Li W Zhang Y. Exploring the complexities of TGF-β signaling in keloids: beyond the classical Smad pathway. Int J Mol Sci. (2026) 27(8). 10.3390/ijms27083600 PMC13116467 42074238

[39] Zhu H Liu X Ouyang W Hao Y Ding Z Tan K. Sfrp1 as a pivotal paracrine factor in the trained pericardial stem cells that foster reparative activity. Stem Cells Transl Med. (2024) 13(2):137–50. 10.1093/stcltm/szad075 37936560 PMC10872698

[40] Sun J Dong M Xiang X Zhang S Wen D. Notch signaling and targeted therapy in non-small cell lung cancer. Cancer Lett. (2024) 585:216647. 10.1016/j.canlet.2024.216647 38301911

[41] Veltrop RJA Kukk MM Topouzidou K Didden L Muchir A van Steenbeek FG. From gene to mechanics: a comprehensive insight into the mechanobiology of LMNA mutations in cardiomyopathy. Cell Commun Signal. (2024) 22(1):197. 10.1186/s12964-024-01546-5 38539233 PMC10976794

[42] Rachedi NS Tang Y Tai YY Zhao J Chauvet C Grynblat J. Dietary intake and glutamine-serine metabolism control pathologic vascular stiffness. Cell Metab. (2024) 36(6):1335–50.e8. 10.1016/j.cmet.2024.04.010 38701775 PMC11152997

[43] Tu Q Gong X Yuan X Tang Y Huang R Jiang Q. NETs drive myocardial fibrosis in hypertension via an NF-κB/ferroptosis axis. Front Immunol. (2025) 16:1712786. 10.3389/fimmu.2025.1712786 41607775 PMC12834794

[44] Liu J Zheng X Zhang C Zhang C Bu P. Lcz696 alleviates myocardial fibrosis after myocardial infarction through the sFRP-1/wnt/β-catenin signaling pathway. Front Pharmacol. (2021) 12:724147. 10.3389/fphar.2021.724147 34539406 PMC8443774

[45] Shumliakivska M Luxán G Hemmerling I Scheller M Li X Müller-Tidow C. DNMT3A clonal hematopoiesis-driver mutations induce cardiac fibrosis by paracrine activation of fibroblasts. Nat Commun. (2024) 15(1):606. 10.1038/s41467-023-43003-w 38242884 PMC10799021

[46] You T Zhang Y Su H Wang N Zhang X Anwaier S. Ameliorating post-infarction myocardial fibrosis and cardiac function via ROS-responsive hydrogel-mediated IL-11 antibody delivery. Bioact Mater. (2026) 60:527–38. 10.1016/j.bioactmat.2026.01.041 41674553 PMC12887659

[47] Zhang X Xiao Y Hu B Li Y Zhang S Tian J. Multi-omics analysis of human tendon adhesion reveals that ACKR1-regulated macrophage migration is involved in regeneration. Bone Res. (2024) 12(1):27. 10.1038/s41413-024-00324-w 38714649 PMC11076548

[48] Vemuri V Kratholm N Nagarajan D Cathey D Abdelbaset-Ismail A Tan Y. Withaferin A as a potential therapeutic target for the treatment of angiotensin II-induced cardiac cachexia. Cells. (2024) 13(9):783. 10.3390/cells13090783 38727319 PMC11083229

[49] Kochumon S Al-Sayyar A Jacob T Bahman F Akhter N Wilson A. TGF-β and TNF-α interaction promotes the expression of MMP-9 through H3K36 dimethylation: implications in breast cancer metastasis. Front Immunol. (2024) 15:1430187. 10.3389/fimmu.2024.1430187 39351229 PMC11439675

[50] Tan Q Gao R Zhang X Yang J Xing P Yang S. Longitudinal plasma proteomic analysis identifies biomarkers and combinational targets for anti-PD1-resistant cancer patients. Cancer Immunol Immunother. (2024) 73(3):47. 10.1007/s00262-024-03631-7 38349411 PMC10864508

[51] Zhu X Huo Q Jin L Du J Yu J Wang H. PARP1 stabilizes FOXN3 to suppress pulmonary fibrosis through p38-related feedback regulation. Sci Adv. (2026) 12(1):eady1681. 10.1126/sciadv.ady1681 41481720 PMC12758542

[52] Duangrat R Parichatikanond W Mangmool S. Dual blockade of TGF-β receptor and endothelin receptor synergistically inhibits angiotensin II-induced myofibroblast differentiation: role of AT(1)R/G(αq)-mediated TGF-β1 and ET-1 signaling. Int J Mol Sci. (2023) 24(8):783. 10.3390/ijms24086972 37108136 PMC10138810

[53] Bagheri L Javanbakht M Malekian S Ghahderijani BH Taghipour S Tanha FD. Antifibrotic therapeutic strategies in systemic sclerosis: critical role of the wnt/β-catenin and TGF-β signal transduction pathways as potential targets. Eur J Pharmacol. (2025) 999:177607. 10.1016/j.ejphar.2025.177607 40209848

[54] Ruta A Krishnan K Woo J Mejías JC Gray-Gaillard EF Maestas DR Jr γδ T cell-stromal networks modulate matrix composition and vascularity in foreign body response. J. Immunol. (2010) 184:6367–77. 10.1038/s41467-026-71540-7 41997939 PMC13272810

[55] Zhou JX Cheng AS Chen L Li LX Agborbesong E Torres VE. CD74 promotes cyst growth and renal fibrosis in autosomal dominant polycystic kidney disease. Cells. (2024) 13(6):489. 10.3390/cells13060489 38534333 PMC10968819

[56] Sun L Zhang HB Jiang HC Li W Li MK Yang XY. LMO7 Drives profibrotic fibroblast polarization and pulmonary fibrosis in mice through TGF-β signalling. Acta Pharmacol Sin. (2025) 46(7):1930–45. 10.1038/s41401-025-01488-9 40000880 PMC12205043

[57] Liu W Ding Z Tao Y Liu S Jiang M Yi F. A positive feedback loop between PFKP and c-Myc drives head and neck squamous cell carcinoma progression. Mol Cancer. (2024) 23(1):141. 10.1186/s12943-024-02051-6 38982480 PMC11232239

[58] Merlo M Zoccatelli F Costa G Panepinto L Friso S Marzano L. Aldosterone synthase inhibitors across the translational spectrum: mechanistic foundations and emerging clinical applications. J Intern Med. (2026) 299(4):425–43. 10.1111/joim.70061 41491887

[59] Yeo TM Chin CWL Seah CWA Cheng LJ Lin W Dalakoti M. Global prevalence of myocardial fibrosis among individuals with cardiometabolic conditions: a systematic review and meta-analysis. Eur J Prev Cardiol. (2025) 32(12):1077–91. 10.1093/eurjpc/zwaf083 39968765

[60] Ren H Wang J Liu J Zhang Z Wang L Wei F. NAD(+)/Nrf2 signaling promotes osteogenesis by regulating oxidative level of BMSCs under mechanical stress. Prog Orthod. (2025) 26(1):19. 10.1186/s40510-025-00566-2 40445451 PMC12125440

[61] Hata K Yanagihara T Matsubara K Kunimura K Suzuki K Tsubouchi K. Mass cytometry identifies characteristic immune cell subsets in bronchoalveolar lavage fluid from interstitial lung diseases. Front Immunol. (2023) 14:1145814. 10.3389/fimmu.2023.1145814 36949950 PMC10027011

[62] Habib YH Khattab M Gowayed MA. The renin-angiotensin system (RAS) and arthritic diseases: therapeutic potential for RAS inhibitors. Inflammopharmacology. (2025) 33(9):5037–47. 10.1007/s10787-025-01890-z 40796991

[63] Caroccia B Caputo I Bertoldi G Favaro V Angelini A Benetti A. Angiotensin II activates yes-associated protein (YAP) in fibroblast promoting deep fascia remodeling. Int J Mol Sci. (2025) 26(22):11105. 10.3390/ijms262211105 41303588 PMC12652227

[64] Arshad MS Saad M Lurz P Rommel KP Khan MS Bangalore S. Intensive blood pressure control in heart failure with preserved ejection fraction: a symptom-modifying strategy. Heart Fail Rev. (2026) 31(1):31. 10.1007/s10741-026-10600-y 41801485

[65] Zhang J Yu H Song X Li X Xie J Wang Y. Chronic kidney disease onset, progression, and cardiovascular outcomes: proteomics informs biology and risk stratification. Cardiovasc Diabetol. (2026) 25(1):51. 10.1186/s12933-025-03049-0 41559658 PMC12903537

[66] Speer T Schunk SJ Sarakpi T Schmit D Wagner M Arnold L. Urinary DKK3 as a biomarker for short-term kidney function decline in children with chronic kidney disease: an observational cohort study. Lancet Child Adolesc Health. (2023) 7(6):405–14. 10.1016/s2352-4642(23)00049-4 37119829

[67] Xu Y Ma C Sun Y Zhu J He S Gao H. Serum proteomics reveals diagnostic biomarkers and molecular pathways in cerebral palsy. Nat Commun. (2025) 16(1):10253. 10.1038/s41467-025-65110-6 41271696 PMC12638935

[68] Rajagopalan S Dobre M Dazard JE Vergara-Martel A Connelly K Farkouh ME. Mineralocorticoid receptor antagonism prevents aortic plaque progression and reduces left ventricular mass and fibrosis in patients with type 2 diabetes and chronic kidney disease: the MAGMA trial. Circulation. (2024) 150(9):663–76. 10.1161/circulationaha.123.067620 39129649 PMC11503525

[69] Du X Duan M Kan S Yang Y Xu S Wei J. TGF-β3 mediates mitochondrial dynamics through the p-Smad3/AMPK pathway. Cell Prolif. (2024) 57(5):e13579. 10.1111/cpr.13579 38012096 PMC11056712

[70] Long M Sun J Zhou D Chen C Shu J Mao X. Lung-derived small extracellular vesicles containing miR-15a-5p/miR-96-5p promote myocardial fibrosis in hypertrophic cardiomyopathy via targeting SMAD7. Small. (2025) 21(46):e01610. 10.1002/smll.202501610 40746011 PMC12632412

[71] Simmonds P Adriaenssens EM Zerbini FM Abrescia NGA Aiewsakun P Alfenas-Zerbini P. Four principles to establish a universal virus taxonomy. PLoS Biol. (2023) 21(2):e3001922. 10.1371/journal.pbio.3001922 36780432 PMC9925010

[72] Lorite P Domínguez JN Palomeque T Torres MI. Extracellular vesicles: advanced tools for disease diagnosis, monitoring, and therapies. Int J Mol Sci. (2024) 26(1):189. 10.3390/ijms26010189 39796048 PMC11720073

[73] Li B Shaikh F Younes H Abuhalimeh B Zamzam A Abdin R. Matrix metalloproteinases 7 and 10 are prognostic biomarkers for systemic cardiovascular risk in individuals with peripheral artery disease. Biomolecules. (2025) 15(6):853. 10.3390/biom15060853 40563493 PMC12190543

[74] D’Amélio F Araldi RP Batista IFC Prieto-da-Silva Á RB Kerkis I. Transcriptomic dissection of bothrops moojeni venom reveals fraction-specific modulation of host cellular pathways. Int J Mol Sci. (2026) 27(13):5943. 10.3390/ijms27135943 PMC13361672 42450210

[75] Marques SS Liebaug A Maurer S Rothenbacher D Brenner RE Riegger J. Growth/differentiation factor 15 promotes a pro-regenerative response in chondrocytes upon cartilage injury. MedComm. (2025) 6(12):e70484. 10.1002/mco2.70484 41287825 PMC12640616

[76] Fridlich O Peretz A Fox-Fisher I Pyanzin S Dadon Z Shcolnik E. Elevated cfDNA after exercise is derived primarily from mature polymorphonuclear neutrophils, with a minor contribution of cardiomyocytes. Cell Rep. Med. (2023) 4(6):101074. 10.1016/j.xcrm.2023.101074 37290439 PMC10313937

[77] Chung J Xiao S Gao Y Soung YH. Recent technologies towards diagnostic and therapeutic applications of circulating nucleic acids in colorectal cancers. Int J Mol Sci. (2024) 25(16). 10.3390/ijms25168703 PMC11354501 39201393

[78] Gao J Zhang X Ding J Zhang H Zhang X Jiang J. The characteristic expression of circulating MicroRNAs in osteoporosis: a systematic review and meta-analysis. Front Endocrinol (Lausanne). (2024) 15:1481649. 10.3389/fendo.2024.1481649 39736860 PMC11682891

[79] Zeng W Liu CC Li S Zhou Y Stackpole ML Xiao Y. Toward the simultaneous detection of multiple diseases with a highly cost-effective cell-free DNA methylome test. Proc Natl Acad Sci U S A. (2026) 123(15):e2518347123. 10.1073/pnas.2518347123 41941615 PMC13080018

[80] Zhang X Sun S Ren G Liu W Chen H. Advances in intercellular communication mediated by exosomal ncRNAs in cardiovascular disease. Int J Mol Sci. (2023) 24(22):16197. 10.3390/ijms242216197 38003385 PMC10671547

[81] Wang L Zhang J He Z Kong X Liu C Xia Y. Exosomal miRNAs in pancreatitis: mechanisms and potential applications (review). Mol Med Rep. (2025) 32(2):1–14. 10.3892/mmr.2025.13575 PMC12131705 40417921

[82] Zeng H Guo S Ren X Wu Z Liu S Yao X. Current strategies for exosome cargo loading and targeting delivery. Cells. (2023) 12(10) :1416. 10.3390/cells12101416 37408250 PMC10216928

[83] Amyar A Nakamori S Ngo L Ishida M Nakamura S Omori T. Cardiovascular magnetic resonance radiologic-pathologic correlation in radiomic analysis of myocardium in non-ischemic dilated cardiomyopathy. J Cardiovasc Magn Reson. (2025) 27(1):101881. 10.1016/j.jocmr.2025.101881 40118277 PMC12138411

[84] Karur GR Aneja A Stojanovska J Hanneman K Latchamsetty R Kersting D. Imaging of cardiac fibrosis: an update, from the AJR special series on imaging of fibrosis. AJR Am J Roentgenol. (2024) 222(6):e2329870. 10.2214/ajr.23.29870 37753860

[85] Kwak S Kim J Jeong MH Park CS Lee HJ Park JB. Integrated use of late gadolinium enhancement and left ventricular global longitudinal strain in hypertrophic cardiomyopathy. JACC Asia. (2026) 6(2):193–205. 10.1016/j.jacasi.2025.07.026 41026067 PMC12904828

[86] Miller RJH Chareonthaitawee P Slomka PJ. Artificial intelligence in nuclear cardiology: enhancing diagnostic accuracy and efficiency. Prog Cardiovasc Dis. (2025) 93:85–91. 10.1016/j.pcad.2025.09.010 41038418

[87] Caldiroli A Capuzzi E Affaticati LM Surace T Di Forti CL Dakanalis A. Candidate biological markers for social anxiety disorder: a systematic review. Int J Mol Sci. (2023) 24(1):835. 10.3390/ijms24010835 36614278 PMC9821596

[88] Duff S Wettersten N Horiuchi Y van Veldhuisen DJ Raturi S Irwin R. Absence of kidney tubular injury in patients with acute heart failure with acute kidney injury. Circ Heart Failure. (2024) 17(11):e011751. 10.1161/circheartfailure.123.011751 39421939 PMC11573103

[89] Ioannou A Patel RK Martinez-Naharro A Razvi Y Porcari A Rauf MU. Tracking treatment response in cardiac light-chain amyloidosis with native T1 mapping. JAMA cardiology. (2023) 8(9):848–52. 10.1001/jamacardio.2023.2010 37466990 PMC10357357

[90] Latiano M De Angelis M Latiano A Palmieri O Latiano TP Delcuratolo MD. Liquid biopsy frontiers in pancreatic cancer: insights from circulating cell-free nucleic acids. Cells. (2026) 15(10):904. 10.3390/cells15100904 42193913 PMC13204079

[91] Yang Y Lv W Shao Y Xu C Feng Y Huang Y. Circular RNA-based molecular computation enhances plasma biomarker detection in biliary tract cancer. Angew Chem Int Ed Engl. (2025) 64(26):e202505289. 10.1002/anie.202505289 40259434

[92] Osokina A Karetnikova V Polikutina O Ivanova A Gruzdeva O Dyleva Y. Prognostic potential of cardiac structural and functional parameters and N-terminal propeptide of type III procollagen in predicting cardiac fibrosis one year after myocardial infarction with preserved left ventricular ejection fraction. Aging. (2021) 13(1):194–203. 10.18632/aging.202495 33431713 PMC7835023

[93] Stienen S Ferreira JP Bär C Thum T Barros A Pitt B. Serum microRNAs and antifibrotic response to eplerenone in acute myocardial infarction complicated by systolic dysfunction. Int J Cardiol. (2021) 332:35–7. 10.1016/j.ijcard.2021.02.088 33676945

[94] Benkhoff M Alde K Ehreiser V Dahlmanns J Metzen D Haurand JM. Thromboinflammation is associated with clinical outcome after ST-elevation myocardial infarction. Blood Adv. (2024) 8(21):5581–9. 10.1182/bloodadvances.2024014273 39226457 PMC11541696

[95] Sarhan N Essam Abou Warda A Alsahali S Alanazi AS. Impact of vitamin D supplementation on the clinical outcomes and epigenetic markers in patients with acute coronary syndrome. Pharmaceuticals (Basel). (2023) 16(2):262. 10.3390/ph16020262 37259407 PMC9967129

[96] Gerstein HC Lee SF Paré G Bethel MA Colhoun HM Hoover A. Biomarker changes associated with both dulaglutide and cardiovascular events in the REWIND randomized controlled trial: a nested case-Control *post hoc* analysis. Diabetes Care. (2023) 46(5):1046–51. 10.2337/dc22-2397 36897834

[97] Trullàs JC Morales-Rull JL Casado J Carrera-Izquierdo M Sánchez-Marteles M Conde-Martel A. Combining loop and thiazide diuretics for acute heart failure across the estimated glomerular filtration rate spectrum: a *post-hoc* analysis of the CLOROTIC trial. Eur J Heart Fail. (2023) 25(10):1784–93. 10.1002/ejhf.2988 37540036

[98] Ravassa S Lupón J López B Codina P Domingo M Revuelta-López E. Prediction of left ventricular reverse remodeling and outcomes by circulating collagen-derived peptides. JACC Heart Failure. (2023) 11(1):58–72. 10.1016/j.jchf.2022.09.008 36599551

[99] Ma C Jia J Lan J Wang J Rao D Rao L. CK2α deficiency drives myocardial fibrosis via desmin-induced mitochondrial dysfunction. Adv Sci (Weinh). (2026) 13(42):e75560. 10.1002/advs.75560 42095508 PMC13336054

[100] Cheng J Bao G Lin D Wang H Yang Y Chen Y. Silk fibroin counteracts fibroblast senescence to restore ECM homeostasis in aged skin. Bioact Mater. (2026) 58:666–84. 10.1016/j.bioactmat.2025.12.006 41551197 PMC12808896

[101] Johnson-Martínez JP Diener C Levine AE Wilmanski T Suskind DL Ralevski A. Aberrant bowel movement frequencies coincide with increased microbe-derived blood metabolites associated with reduced organ function. Cell Rep Med. (2024) 5(7):101646. 10.1016/j.xcrm.2024.101646 PMC11293344 39019013

[102] Kim JY Gil TH Lee HG Shin JW Jang DH Kim HS. Plasma extracellular vesicles biomarkers linked to lower muscle mass, function and physical performance in sarcopenia. J Cachexia Sarcopenia Muscle. (2025) 16(2):e13784. 10.1002/jcsm.13784 40162588 PMC11955922

[103] Revnic R Cojan-Minzat BO Zlibut A Orzan RI Agoston R Muresan ID. The role of circulating collagen turnover biomarkers and late gadolinium enhancement in patients with non-ischemic dilated cardiomyopathy. Diagnostics (Basel). (2022) 12(6):1435. 10.3390/diagnostics12061435 35741245 PMC9222171

[104] Krebber MM Van Dijk CGM Vernooij RWM Brandt MM Emter CA Rau CD. Matrix metalloproteinases and tissue inhibitors of metalloproteinases in extracellular matrix remodeling during left ventricular diastolic dysfunction and heart failure with preserved ejection fraction: a systematic review and meta-analysis. Bioinformatics. (2011) 27:1739–40. 10.3390/ijms21186742 32937927 PMC7555240

[105] Liu H Yan W Ma C Zhang K Li K Jin R. Early detection of cardiac fibrosis in diabetic mice by targeting myocardiopathy and matrix metalloproteinase 2. Acta Biomater. (2024) 176:367–78. 10.1016/j.actbio.2024.01.017 38244659

[106] Çelik Ö Şahin AA Sarıkaya S Uygur B. Correlation between serum matrix metalloproteinase and myocardial fibrosis in heart failure patients with reduced ejection fraction: a retrospective analysis. Anatol J Cardiol. (2020) 24(5):303–8. 10.14744/AnatolJCardiol.2020.54937 PMC7724386 33122477

[107] Truong VT Moore RA Lubert AM Taylor MD Mazur W Alsaied T. Association of plasma biomarkers and interstitial myocardial fibrosis in fontan population: a machine learning approach. Int J Cardiol Congenit Heart Dis. (2022) 7:100321. 10.1016/j.ijcchd.2022.100321 39712285 PMC11657535

[108] Yang Y Ma L Wang C Song M Li C Chen M. Matrix metalloproteinase-7 in platelet-activated macrophages accounts for cardiac remodeling in uremic mice. Basic Res Cardiol. (2020) 115(3):30. 10.1007/s00395-020-0789-z 32270301

[109] Ye Q Liu Q Ma X Bai S Chen P Zhao Y. MicroRNA-146b-5p promotes atrial fibrosis in atrial fibrillation by repressing TIMP4. J Cell Mol Med. (2021) 25(22):10543–53. 10.1111/jcmm.16985 34643044 PMC8581305

[110] Tan W Li X Zheng S Li X Zhang X Pyle WG. A porcine model of heart failure with preserved ejection fraction induced by chronic pressure overload characterized by cardiac fibrosis and remodeling. Front Cardiovasc Med. (2021) 8:677727. 10.3389/fcvm.2021.677727 34150870 PMC8206269

[111] Ridwan M Dimiati H Syukri M Lesmana R. Potential molecular mechanism underlying cardiac fibrosis in diabetes mellitus: a narrative review. Egypt Heart J. (2023) 75(1):46. 10.1186/s43044-023-00376-z 37306727 PMC10260731

[112] Su C Wang Q Luo H Jiao W Tang J Li L. Si-Miao-Yong-An decoction attenuates cardiac fibrosis via suppressing TGF-β1 pathway and interfering with MMP-TIMPs expression. Biomed Pharmacother. (2020) 127:110132. 10.1016/j.biopha.2020.110132 32403042

[113] Grillet B Pereira RVS Van Damme J Abu El-Asrar A Proost P Opdenakker G. Matrix metalloproteinases in arthritis: towards precision medicine. Nat Rev Rheumatol. (2023) 19(6):363–77. 10.1038/s41584-023-00966-w 37161083

[114] Aksnes M Edwin TH Saltvedt I Eldholm RS Chaudhry FA Halaas NB. Sex-specific associations of matrix metalloproteinases in Alzheimer's disease. Biol Sex Differ. (2023) 14(1):35. 10.1186/s13293-023-00514-x 37221606 PMC10207710

[115] Blanda V Bracale UM Di Taranto MD Fortunato G. Galectin-3 in cardiovascular diseases. Int J Mol Sci. (2020) 21(23):9232. 10.3390/ijms21239232 33287402 PMC7731136

[116] Spoladore R Falasconi G Fiore G Di Maio S Preda A Slavich M. Cardiac fibrosis: emerging agents in preclinical and clinical development. Expert Opin Invest Drugs. (2021) 30(2):153–66. 10.1080/13543784.2021.1868432 33356660

[117] Zaborska B Sikora-Frąc M Smarż K Pilichowska-Paszkiet E Budaj A Sitkiewicz D. The role of galectin-3 in heart failure-the diagnostic, prognostic and therapeutic potential-where do we stand? Int J Mol Sci. (2023) 24(17):13111. 10.3390/ijms241713111 37685918 PMC10488150

[118] Fernandes F Moreira CHV Oliveira LC Souza-Basqueira M Ianni BM Lorenzo CD. Galectin-3 associated with severe forms and long-term mortality in patients with Chagas disease. Arq Bras Cardiol. (2021) 116(2):248–56. 10.36660/abc.20190403 33656072 PMC7909980

[119] Visoiu IS Rimbas RC Nicula AI Mihaila-Baldea S Magda SL Mihalcea DJ. Multimodality imaging and biomarker approach to characterize the pathophysiology of heart failure in left ventricular non-compaction with preserved ejection fraction. J Clin Med. (2023) 12(11):3632. 10.3390/jcm12113632 37297827 PMC10253280

[120] Niu J Feng F Zhang S Zhu Y Song R Li J. Thrombospondin-2 couples pressure-promoted chondrogenesis through NF-κB signaling. Tissue Eng Regen Med. (2023) 20(5):753–66. 10.1007/s13770-023-00548-7 37219820 PMC10352201

[121] Ratziu V Surabattula R Bugianesi E Schattenberg JM Myneni S Rosso C. Thrombospondin-2 is a performant biomarker of at-risk MASH and advanced MASH fibrosis in a large multicentre European cohort. Gut. (2026) 75(6):1226–36. 10.1136/gutjnl-2025-335598 40738743

[122] Saffi H Winsløw U Sakthivel T Højgaard EV Linde J Philbert B. Global constructive work is associated with ventricular arrhythmias after cardiac resynchronization therapy. Eur Heart J Cardiovasc Imaging. (2023) 25(1):29–36. 10.1093/ehjci/jead180 37490039

[123] Xie X Zhang Y Fang Y Wu J Li Q. Molecular basis of high-blood-pressure-enhanced and high-fever-temperature-weakened receptor-binding domain/peptidase domain binding: a molecular dynamics simulation study. Int J Mol Sci. (2025) 26(7):3250. 10.3390/ijms26073250 40244099 PMC11989460

[124] Zhou X Zhang L Lin X Chen X Liu H Yuan X. Thrombospondin 2 is a novel biomarker of essential hypertension and associated with nocturnal na(+) excretion and insulin resistance. Clin Exp Hypertens (New York). (2023) 45(1):2276029. 10.1080/10641963.2023.2276029 37943619

[125] Bouffette S Botez I De Ceuninck F. Targeting galectin-3 in inflammatory and fibrotic diseases. Trends Pharmacol Sci. (2023) 44(8):519–31. 10.1016/j.tips.2023.06.001 37391294

[126] Otaki Y Shimizu M Watanabe T Tachibana S Sato J Kobayashi Y. Growth differentiation factor 15 and clinical outcomes in Japanese patients with heart failure. Circ J. (2023) 87(8):1120–9. 10.1253/circj.CJ-23-0088 36948614

[127] Rochette L Dogon G Zeller M Cottin Y Vergely C. GDF15 And cardiac cells: current concepts and new insights. Int J Mol Sci. (2021) 22(16):8889. 10.3390/ijms22168889 34445593 PMC8396208

[128] Mohammed HH Ahmed FM Ahmed SN. Growth differentiation factor-15 as a predictor of acute myocardial infarction: a multivariable modeling approach. Monaldi Arch Chest Dis. (2025) 3426. 10.4081/monaldi.2025.3426 41384393

[129] Wereski R Kimenai DM Taggart C Doudesis D Lee KK Lowry MTH. Cardiac troponin thresholds and kinetics to differentiate myocardial injury and myocardial infarction. Circulation. (2021) 144(7):528–38. 10.1161/circulationaha.121.054302 34167318 PMC8360674

[130] Lombardi C Marandola M Loria V Urbani A Baroni S. Growth differentiation factor-15 as an emerging biomarker in cardiology: diagnostic and prognostic implications. J Pers Med. (2026) 16(1):16. 10.3390/jpm16010016 41590509 PMC12843272

[131] Scisciola L Paolisso P Belmonte M Gallinoro E Delrue L Taktaz F. Myocardial sodium-glucose cotransporter 2 expression and cardiac remodelling in patients with severe aortic stenosis: the BIO-AS study. Eur J Heart Fail. (2024) 26(2):471–82. 10.1002/ejhf.3145 38247224

[132] Sawant H Borthakur A. Disease-specific novel role of growth differentiation factor 15 in organ fibrosis. Int J Mol Sci. (2025) 26(12):5713. 10.3390/ijms26125713 40565178 PMC12193308

[133] Guo H Zhao X Li H Liu K Jiang H Zeng X. GDF15 promotes cardiac fibrosis and proliferation of cardiac fibroblasts via the MAPK/ERK1/2 pathway after irradiation in rats. Radiat Res. (2021) 196(2):183–91. 10.1667/rade-20-00206.1 34019665

[134] Kanagala P Arnold JR Singh A Chan DCS Cheng ASH Khan JN. Characterizing heart failure with preserved and reduced ejection fraction: an imaging and plasma biomarker approach. PLoS One. (2020) 15(4):e0232280. 10.1371/journal.pone.0232280 32349122 PMC7190371

[135] Wimalanathan T Paus MF Brox Skranes J Berge T Tveit A Røsjø H. Associations between growth differentiation factor 15, cardiac troponin T, and N-terminal pro-B-type natriuretic peptide, and future myocardial fibrosis assessed by cardiac magnetic resonance imaging: data from the Akershus Cardiac Examination 1950 Study. J Appl Lab Med. (2025) 10(2):392–405. 10.1093/jalm/jfae145 39707823

[136] Nabi-Afjadi M Farzam F Soltani S Golestani N Asl SS Aziziyan F. The association between periostin and tumor microenvironment: a promising cancer prognostic biomarker and therapeutic target to combat tumor progression and chemoresistance. Cancer Cell Int. (2025) 25(1):320. 10.1186/s12935-025-03959-9 40903761 PMC12406393

[137] Giarratana AO Prendergast CM Salvatore MM Capaccione KM. TGF-β signaling: critical nexus of fibrogenesis and cancer. J Transl Med. (2024) 22(1):594. 10.1186/s12967-024-05411-4 38926762 PMC11201862

[138] Miao Q Hua S Gong Y Lyu Z Qian P Liu C. Free-breathing non-contrast T1ρ dispersion magnetic resonance imaging of myocardial interstitial fibrosis in comparison with extracellular volume fraction. J Cardiovasc Magn Reson. (2024) 26(2):101093. 10.1016/j.jocmr.2024.101093 39245148 PMC11612770

[139] Hua Y Lu H Wang S Yue X Du F Zhang N. Deep learning reconstruction for fast cardiovascular magnetic resonance imaging protocol: a comparative study with conventional cardiovascular magnetic resonance. J Cardiovasc Magn Resone. (2026) 28(1):102017. 10.1016/j.jocmr.2025.102017 PMC12811447 41318031

[140] Fillingim M Tanguay-Sabourin C Parisien M Zare A Guglietti GV Norman J. Biological markers and psychosocial factors predict chronic pain conditions. Nat Hum Behav. (2025) 9(8):1710–25. 10.1038/s41562-025-02156-y 40355673 PMC12367528

[141] Hori M Takahashi H Kondo C Takeda A Morozumi K Maruyama S. High serum ferritin levels are associated with sarcopenia in patients undergoing chronic hemodialysis. Nutrients. (2025) 17(14):2323. 10.3390/nu17142323 40732948 PMC12298234

[142] Xiang JY Dai YS Zheng JY Yu LY Hu J Song A. Cardiac magnetic resonance-derived extracellular volume radiomics in reperfused ST-elevation myocardial infarction: long-term prognostic value and risk stratification. Eur Heart J Cardiovasc Imaging. (2025) 26(8):1376–86. 10.1093/ehjci/jeaf140 40338050

[143] Armandi A Sanavia T Younes R Caviglia GP Rosso C Govaere O. Serum ferritin levels can predict long-term outcomes in patients with metabolic dysfunction-associated steatotic liver disease. Gut. (2024) 73(5):825–34. 10.1136/gutjnl-2023-330815 38199805

[144] Yasuda N Kato S Horita N Sekii R Sawamura S Nagase H. Synthetic extracellular volume fraction as an imaging biomarker of the myocardial interstitium without blood sampling: a systematic review and meta-analysis. J Cardiovasc Magn Reson. (2025) 27(1):101889. 10.1016/j.jocmr.2025.101889 40139292 PMC12138551

[145] González A López B Ravassa S San José G Latasa I Butler J. Myocardial interstitial fibrosis in hypertensive heart disease: from mechanisms to clinical management. Hypertension (Dallas). (2024) 81(2):218–28. 10.1161/hypertensionaha.123.21708 38084597

[146] Yu S Chen X Dong Z Cheng H Yang K Yin G. T1 mapping for identifying the substrate in patients with apparently idiopathic premature ventricular complexes. JACC Clin Electrophysiol. (2023) 9(6):751–61. 10.1016/j.jacep.2022.12.012 37380310

[147] Karabinowska-Małocha A Dziewięcka E Banyś P Urbańczyk-Zawadzka M Krupiński M Mielnik M. The relationship between cardiac magnetic resonance-assessed replacement and interstitial fibrosis and ventricular arrhythmias in hypertrophic cardiomyopathy. J Pers Med. (2022) 12(2):294. 10.3390/jpm12020294 PMC8876292 35207782

[148] Chen YL Chen CH Ye XF Xu JZ Zhu LM Xu TY. Myocardial fibrosis in primary aldosteronism. Front Endocrinol (Lausanne). (2025) 16:1567876. 10.3389/fendo.2025.1567876 40395817 PMC12088938

[149] Zhi Y Zhang TY Gui FD Wen M Gao LC Long YT. Myocardial fibrosis evaluated by T1 mapping and its relationship to left ventricular hypertrophy, strain, and T2 value in hypertrophic cardiomyopathy without late gadolinium enhancement. J Thorac Imaging. (2025) 41(3). 10.1097/rti.0000000000000862 41084187

[150] Boros GAB Hueb W Rezende PC Rochitte CE Nomura CH Lima EG. Unveiling myocardial microstructure shifts: exploring the impact of diabetes in stable CAD patients through CMR T1 mapping. Diabetol Metab Syndr. (2024) 16(1):156. 10.1186/s13098-024-01395-9 38982515 PMC11232262

[151] Yang P Dong X Zhang Y. MicroRNA profiles in plasma samples from young metabolically healthy obese patients and miRNA-21 are associated with diastolic dysfunction via TGF-β1/Smad pathway. J Clin Lab Anal. (2020) 34(6):e23246. 10.1002/jcla.23246 32108968 PMC7307369

[152] Yuan Y Xiao Y Zhao J Zhang L Li M Luo L. Exosomes as novel biomarkers in sepsis and sepsis related organ failure. J Transl Med. (2024) 22(1):1078. 10.1186/s12967-024-05817-0 39609831 PMC11604007

[153] Chang M Jiang S Guo X Gao J Liu P Bao X. Exosomal RNAs in the development and treatment of pituitary adenomas. Front Endocrinol (Lausanne). (2023) 14:1142494. 10.3389/fendo.2023.1142494 36875488 PMC9981947

[154] Son CJ Carnino JM Lee H Jin Y. Emerging roles of circular RNA in macrophage activation and inflammatory lung responses. Cells. (2024) 13(17):1407. 10.3390/cells13171407 39272979 PMC11394395

[155] Abdelgawad A Huang Y Gololobova O Yu Y Witwer KW Parashar V. Defining the parameters for sorting of RNA cargo into extracellular vesicles. J Extracell Vesicles. (2025) 14(7):e70113. 10.1002/jev2.70113 40620070 PMC12230367

[156] Liu L Lei X Wang Z Meng J Song B. TransRM: weakly supervised learning of translation-enhancing N6-methyladenosine (m(6)A) in circular RNAs. Int J Biol Macromol. (2025) 306(Pt 2):141588. 10.1016/j.ijbiomac.2025.141588 40023417

[157] Nejad A E Zadeh SM M Nickho H Sadoogh Abbasian A Forouzan A Ahmadlou M. The role of microRNAs involved in the disorder of blood-brain barrier in the pathogenesis of multiple sclerosis. Front Immunol. (2023) 14:1281567. 10.3389/fimmu.2023.1281567 38193092 PMC10773759

[158] Li F Long TY Bi SS Sheikh SA Zhang CL. circPAN3 exerts a profibrotic role via sponging miR-221 through FoxO3/ATG7-activated autophagy in a rat model of myocardial infarction. Life Sci. (2020) 257:118015. 10.1016/j.lfs.2020.118015 32629000

[159] Han J Zhang Y Ge P Dakal TC Wen H Tang S. Exosome-derived CIRP: an amplifier of inflammatory diseases. Front Immunol. (2023) 14:1066721. 10.3389/fimmu.2023.1066721 36865547 PMC9971932

[160] Kou S Lu Z Deng D Ye M Sui Y Qin L. Activation of imprinted gene PW1 promotes cardiac fibrosis after ischemic injury. Circulation. (2025) 151(9):623–39. 10.1161/circulationaha.124.070738 39704066

[161] Sisson TH Osterholzer JJ Leung L Basrur V Nesvizhskii A Subbotina N. PAI-1 interaction with sortilin-related receptor 1 is required for lung fibrosis. JCI insight. (2025) 10(11):e186131. 10.1172/jci.insight.186131 40299646 PMC12220977

[162] Martinez-Zalbidea I Rzasa A Puvanesarajah V Hitzl W Wuertz-Kozak K. TSG-6 activated MSC-derived extracellular vesicles present altered micro-RNA contents and ameliorate the inflammatory phenotype of macrophages *in vitro*. Inflammation. (2026) 49(1):42. 10.1007/s10753-025-02398-y 41526541 PMC12862027

[163] Mason JD Marks E Fan SJ McCormick K Wilson C Harris AL. Stress-induced Rab11a-exosomes induce amphiregulin-mediated cetuximab resistance in colorectal cancer. J Extracell Vesicles. (2024) 13(6):e12465. 10.1002/jev2.12465 38887984 PMC11184284

[164] Samuels M Karakostas C Besta S Lauer Betrán A Tsilingiri K Turner C. LMTK3 Regulation of EV biogenesis and cargo sorting promotes tumour growth by reducing monocyte infiltration and driving pro-tumourigenic macrophage polarisation in breast cancer. Mol Cancer. (2025) 24(1):149. 10.1186/s12943-025-02346-2 40405280 PMC12100856

[165] Zhang C Huo ST Wu Z Chen L Wen C Chen H. Rapid development of targeting circRNAs in cardiovascular diseases. Mol Ther Nucleic Acids. (2020) 21:568–76. 10.1016/j.omtn.2020.06.022 32721877 PMC7390851

[166] Mallaredy V Roy R Cheng Z Thej C Benedict C Truongcao M. Tipifarnib reduces extracellular vesicles and protects from heart failure. Circ Res. (2024) 135(2):280–97. 10.1161/circresaha.123.324110 38847080 PMC11223950

[167] Li H Shadrin I Helfer A Heman K Rao L Curtis C. In vitro vascularization improves *in vivo* functionality of human engineered cardiac tissues. Acta Biomater. (2026) 211:61–73. 10.1016/j.actbio.2024.11.014 39528062 PMC12064791

[168] Lunde IG Rypdal KB Van Linthout S Diez J González A. Myocardial fibrosis from the perspective of the extracellular matrix: mechanisms to clinical impact. Matrix Biol. (2024) 134:1–22. 10.1016/j.matbio.2024.08.008 39214156

[169] Li H Liu T Yang Y Cho WC Flynn RJ Harandi MF. Interplays of liver fibrosis-associated microRNAs: molecular mechanisms and implications in diagnosis and therapy. Genes Dis. (2023) 10(4):1457–69. 10.1016/j.gendis.2022.08.013 37397560 PMC10311052

[170] Bracken CP Goodall GJ Gregory PA. RNA regulatory mechanisms controlling TGF-β signaling and EMT in cancer. Semin Cancer Biol. (2024) 102-103:4–16. 10.1016/j.semcancer.2024.06.001 38917876

[171] Wang H Ding W Zhang L Xu M Sui J. Transcriptomic identification of diagnostic biomarkers for alcohol-associated liver cirrhosis: integration of population-level epidemiology with multi-cohort transcriptomic analysis. Int J Mol Sci. (2026) 27(13):5809. 10.3390/ijms27135809 42450082 PMC13360815

[172] Liu Y Zhang Y Li H Hu TY. Recent advances in the bench-to-bedside translation of cancer nanomedicines. Acta Pharm Sin B. (2025) 15(1):97–122. 10.1016/j.apsb.2024.12.007 40041906 PMC11873642

[173] Massoud G Parish M Hazimeh D Moukarzel P Singh B Cayton Vaught KC. Unlocking the potential of tranilast: targeting fibrotic signaling pathways for therapeutic benefit. Int Immunopharmacol. (2024) 137:112423. 10.1016/j.intimp.2024.112423 38861914 PMC11245748

[174] Bakhshi H Michelhaugh SA Bruce SA Seliger SL Qian X Ambale Venkatesh B. Association between proteomic biomarkers and myocardial fibrosis measured by MRI: the multi-ethnic study of atherosclerosis. EBioMedicine. (2023) 90:104490. 10.1016/j.ebiom.2023.104490 36857966 PMC10006438

[175] Yongzhong C Hui C Luting Z Wei G Yiqing H Yiru G. Mechanism of jianxin granules in the treatment of heart failure based on proteomics and metabolomics. Chin Med. (2024) 19(1):165. 10.1186/s13020-024-01009-6 39605071 PMC11604013

[176] Tang TT Zhang YL Crowley SD Lv LL Liu BC. Shedding light on the role of extracellular vesicles in renal fibrosis. Fundam Res. (2026) 6(2):1161–70. 10.1016/j.fmre.2023.12.022 41971816 PMC13069652

[177] Gong L Lu X Ma N Lu T Gong Y Hao L. Targeting fibroblast activation protein-α to treat renal fibrosis. Cell Mol Biol Lett. (2026) 31(1):71. 10.1186/s11658-026-00898-9 41906119 PMC13154437

[178] Nassal DM Shaheen R Patel NJ Yu J Leahy N Bibidakis D. Spectrin-based regulation of cardiac fibroblast cell-cell communication. Cells. (2023) 12(5):748. 10.3390/cells12050748 36899883 PMC10001335

[179] Palumbos SD Popolow J Goldsmith J Holzbaur ELF. Autophagic stress activates distinct compensatory secretory pathways in neurons. Proc Natl Acad Sci U S A. (2025) 122(28):e2421886122. 10.1073/pnas.2421886122 40623183 PMC12280970

[180] Chen D Feng Y Wu J Zhou J Li Z Qiao M. Post-translational modifications in mammalian folliculogenesis and ovarian pathologies. Cells. (2025) 14(16):1292. 10.3390/cells14161292 40862771 PMC12385027

[181] Fernandez AI Gaule P Rimm DL. Tissue age affects antigenicity and scoring for the 22C3 immunohistochemistry companion diagnostic test. Mod Pathol. (2023) 36(7):100159. 10.1016/j.modpat.2023.100159 36925070 PMC10502188

[182] Mukherjee MM Pramanik A Kolodrubetz M Biesbrock D Jacobson KA Hanover JA. Perturbing O-GlcNAcase modulates the expression and distribution of galectin-3. Cells. (2026) 15(13):1181. 10.3390/cells15131181 42439658 PMC13360182

[183] Giordano E Liori B Cecchini I Verani R Leone L. In-house CHO HCPs platform: a promising approach for HCPs ELISA monitoring. Eur J Pharm Sci. (2024) 192:106656. 10.1016/j.ejps.2023.106656 38029932

[184] Khoddam A Kalousdian A Eren M Soberanes S Decker A Lux EJ. Plasminogen activator inhibitor 1 promotes aortic aging-like pathophysiology in humans and mice. J Clin Invest. (2025) 135(23):e196714. 10.1172/jci196714 41026613 PMC12646655

[185] Suzuki H Iwamoto H Seki T Nakamura T Masuda A Sakaue T. Tumor-derived insulin-like growth factor-binding protein-1 contributes to resistance of hepatocellular carcinoma to tyrosine kinase inhibitors. Cancer Commun (Lond). (2023) 43(4):415–34. 10.1002/cac2.12411 36825684 PMC10091105

[186] Zhang M Wu X Wen Y Li Z Chen F Zou Y. Epirubicin induces cardiotoxicity through disrupting ATP6V0A2-dependent lysosomal acidification and triggering ferroptosis in cardiomyocytes. Cell Death Discov. (2024) 10(1):337. 10.1038/s41420-024-02095-z 39048556 PMC11269639

[187] Chang CH Nguyen PA Huang CC Liu CF Melisa S Chen CJ. Acute myocardial infarction risk prediction in emergency chest pain patients: an external validation study. Int J Med Inf. (2025) 193:105683. 10.1016/j.ijmedinf.2024.105683 39504915

[188] Xie S Sun Y Zhao X Xiao Y Zhou F Lin L. An update of the molecular mechanisms underlying anthracycline induced cardiotoxicity. Front Pharmacol. (2024) 15:1406247. 10.3389/fphar.2024.1406247 38989148 PMC11234178

[189] Chen Y Zhang Z Cheng Y Chen X Zhou J Wei Z. Fibroblast circSamd4 promotes cardiac fibrosis via activating plasminogen activator inhibitor-1. Redox Biol. (2026) 90:104018. 10.1016/j.redox.2026.104018 41520563 PMC12824917

[190] Nousis L Kanavaros P Barbouti A. Oxidative stress-induced cellular senescence: is labile iron the connecting link? Antioxidants (Basel). (2023) 12(6):1250. 10.3390/antiox12061250 37371980 PMC10295026

[191] Wohlfromm F Seyrek K Ivanisenko N Troitskaya O Kulms D Richter V. RL2 Enhances the elimination of breast cancer cells by doxorubicin. Cells. (2023) 12(24):2779. 10.3390/cells12242779 38132099 PMC10741759

[192] Moreno-Parro I Diaz-Garzon J Aarsand AK Sandberg S Aikin R Equey T. Biological variation data in triathletes for metabolism and growth-related biomarkers included in the athlete biological passport. Clin Chem. (2024) 70(7):987–96. 10.1093/clinchem/hvae072 38781424

[193] Lim SH Yee GT Khang D. Nanoparticle-based combinational strategies for overcoming the blood-brain barrier and blood-tumor barrier. Int J Nanomed. (2024) 19:2529–52. 10.2147/ijn.S450853 PMC10949308 38505170

[194] Dai S Zhao L Wang G Chen C Li C Xiao B. Celiac ganglia neurolysis suppresses high blood pressure in rats. Hypertens Res. (2023) 46(7):1771–81. 10.1038/s41440-023-01305-y 37173429

[195] Ljungdahl A Sanders SJ. Neuropsychiatric biomarker discovery: go big or go home. Trends Mol Med. (2023) 29(11):878–9. 10.1016/j.molmed.2023.09.002 37714797 PMC10879988

[196] Zhao J Ruan Y Chen Y Chen Q Hong T Wang L. Urea cycle fumarate limits fibrosis post-myocardial infarction by reducing fibroblast mitochondrial adenosine triphosphate production. Cardiovasc Res. (2026) 122(10):1359–73. 10.1093/cvr/cvag119 42210031 PMC13355841

[197] Wu X Wang Z Liang Z Li N Chen J Liu Q. Pleiotropic role of CCR9/CCL25 signaling in Adriamycin-induced cardiomyopathy. J Adv Res. (2025) 75:707–22. 10.1016/j.jare.2024.10.018 39442876 PMC12789724

[198] Chen H Xu J Huang Q Zhao J Hu Y Wang C. Kif23 promotes myocardial fibrosis by suppressing Ces1d-dependent lipid metabolism. Hypertension (Dallas). (2026) 83(1):116–29. 10.1161/hypertensionaha.125.25744 41078122

[199] Kanji R Gue YX Memtsas V Spencer NH Gorog DA. Biomarkers of thrombotic status predict spontaneous reperfusion in patients with ST-segment elevation myocardial infarction. J Am Coll Cardiol. (2023) 81(19):1918–32. 10.1016/j.jacc.2023.03.388 37164525

[200] Boichenko V Frolova S Voellenkle C Zaccagnini G Ferrero P Bassareo PP. Unifying and unique roles of non-coding RNA biomarkers in liver and heart fibrosis. Biomed Pharmacother. (2026) 201:119607. 10.1016/j.biopha.2026.119607 42308919 PMC13380601

[201] Zhang SN Guo JJ Zhao LS Bo YK Yang D Yang XM. Integrative proteomics and metabolomics reveal that Terrestris saponin D ameliorates pulmonary fibrosis by regulating unsaturated fatty acid biosynthesis. Phytomedicine. (2025) 148:157232. 10.1016/j.phymed.2025.157232 40957363

[202] Divsalar E İncili GK Shi C Semsari E Hosseini SH Ebrahimi Tirtashi F. Challenges with the use of postbiotics/parabiotics in food industry. Crit Rev Food Sci Nutr. (2026) 66(6):1155–71. 10.1080/10408398.2025.2541047 40747876

[203] Brennan L De Roos B. Role of metabolomics in the delivery of precision nutrition. Redox Biol. (2023) 65:102808. 10.1016/j.redox.2023.102808 37423161 PMC10461186

[204] Li Z Hu Y Zou F Gao W Feng S Chen G. Assessing the risk of TB progression: advances in blood-based biomarker research. Microbiol Res. (2025) 292:128038. 10.1016/j.micres.2024.128038 39752806

[205] Welsh JA Goberdhan DCI O’Driscoll L Buzas EI Blenkiron C Bussolati B. Minimal information for studies of extracellular vesicles (MISEV2023): from basic to advanced approaches. J Extracell Vesicles. (2024) 13(2):e12404. 10.1002/jev2.12404 38326288 PMC10850029

[206] Kim KH Yoo BC. Membrane protein glycosylation revisited: functional dynamics and emerging clinical insights. Int J Mol Sci. (2026) 27(8):3575. 10.3390/ijms27083575 42074215 PMC13115774

[207] Benatar M Macklin EA Malaspina A Rogers ML Hornstein E Lombardi V. Prognostic clinical and biological markers for amyotrophic lateral sclerosis disease progression: validation and implications for clinical trial design and analysis. EBioMedicine. (2024) 108:105323. 10.1016/j.ebiom.2024.105323 39270623 PMC11415817

[208] Tay JK Narasimhan B Hastie T. Elastic net regularization paths for all generalized linear models. J Stat Softw. (2023) 106:i01. 10.18637/jss.v106.i01 PMC10153598 37138589

[209] Zhao Y Lei T Ge X Fan L He Y Yu Z. The mechanobiology of extracellular matrix: a focus on thrombospondins. Cell Commun Signal. (2025) 23(1):354. 10.1186/s12964-025-02365-y 40713624 PMC12291261

[210] Yao Y Feng L Sun Y Wang S Sun J Hu B. Myocardial fibrosis combined with NT-proBNP improves the accuracy of survival prediction in ADHF patients. BMC Cardiovasc Disord. (2021) 21(1):264. 10.1186/s12872-021-02083-6 34049488 PMC8164226

[211] Mannil M Eberhard M von Spiczak J Heindel W Alkadhi H Baessler B. Artificial intelligence and texture analysis in cardiac imaging. Curr Cardiol Rep. (2020) 22(11):131. 10.1007/s11886-020-01402-1 32910325

[212] Challa A Maras JS Nagpal S Tripathi G Taneja B Kachhawa G. Multi-omics analysis identifies potential microbial and metabolite diagnostic biomarkers of bacterial vaginosis. J Eur Acad Dermatol Venereol. (2024) 38(6):1152–65. 10.1111/jdv.19805 38284174

[213] Hong W Zhang Y Wang S Zheng D Hsu S Zhou J. Deciphering the immune modulation through deep transcriptomic profiling and therapeutic implications of DNA damage repair pattern in hepatocellular carcinoma. Cancer Lett. (2024) 582:216594. 10.1016/j.canlet.2023.216594 38135208

[214] Zou S Khoo BL. Subtyping based on immune cell fractions reveal heterogeneity of cardiac fibrosis in end-stage heart failure. Front Immunol. (2023) 14:1053793. 10.3389/fimmu.2023.1053793 36875078 PMC9975711

[215] He M Liu Y Li T Liu Y Wang X Xie J. Machine learning-powered discovery of a novel berberine derivative inducing SCD-dependent ferroptosis in osteosarcoma. J Transl Med. (2025) 23(1):1328. 10.1186/s12967-025-07358-6 41267108 PMC12636167

[216] Chan JH Grace C Mazidi M Clarke R Ho CY Neubauer S. Biobank-Scale plasma proteomics identifies novel biomarkers in hypertrophic cardiomyopathy. Circ Genom Precis Med. (2026) 19(3):e005325. 10.1161/circgen.125.005325 42017215 PMC13263041

[217] Girerd N Levy D Duarte K Ferreira JP Ballantyne C Collier T. Protein biomarkers of new-onset heart failure: insights from the Heart Omics and Ageing Cohort, the Atherosclerosis risk in Communities Study, and the Framingham Heart Study. Circ Heart Fail. (2023) 16(5):e009694. 10.1161/circheartfailure.122.009694 37192292 PMC10179982

[218] Weberling LD Ochs A Benovoy M. Aus dem Siepen F Salatzki J Giannitsis E. Machine learning to automatically differentiate hypertrophic cardiomyopathy, cardiac light chain, and cardiac transthyretin amyloidosis: a multicenter CMR study. Circ Cardiovasc Imaging. (2025) 18(7):017761. 10.1161/circimaging.124.017761 40464070

[219] Kim YT Kim H So M Kong J Kim KT Hong JH. Differentiating loss of consciousness causes through artificial intelligence-enabled decoding of functional connectivity. NeuroImage. (2024) 297:120749. 10.1016/j.neuroimage.2024.120749 39033787

[220] Sinha S Hassan N Schwartz RE. Organelle stress and alterations in interorganelle crosstalk during liver fibrosis. Hepatology (Baltimore). (2024) 79(2):482–501. 10.1097/hep.0000000000000012 36626634

[221] Cantaş Türkiş F. Explainable machine learning for the prediction of Alzheimer's disease-related cognitive impairment: a consensus feature selection approach. BMC Med Inform Decis Mak. (2026) 26(1):328. 10.1186/s12911-026-03585-z PMC13417844 42215935

[222] EvansSR PennelloG ZhangS LiY WangY CaoQ. Intention-to-diagnose and distinct research foci in diagnostic accuracy studies. Lancet Infect Dis. (2025) 25(8):e472–81. 10.1016/s1473-3099(25)00070-240158520

[223] Lin PK Davis GE. Extracellular matrix remodeling in vascular disease: defining its regulators and pathological influence. Arterioscler Thromb Vasc Biol. (2023) 43(9):1599–616. 10.1161/atvbaha.123.318237 37409533 PMC10527588

[224] Amacker A Peng CC Jiang N Sirivolu S Higa N Stachelek K. Phenotypic biomarkers of aqueous extracellular vesicles from retinoblastoma eyes. Int J Mol Sci. (2024) 25(21):11660. 10.3390/ijms252111660 39519212 PMC11545953

[225] Ungvari A Nyúl-Tóth Á Patai R Csik B Gulej R Nagy D. Cerebromicrovascular senescence in vascular cognitive impairment: does accelerated microvascular aging accompany atherosclerosis? GeroScience. (2025) 47(4):5511–24. 10.1007/s11357-025-01621-w 40113668 PMC12397478

[226] Chehab O Abdollahi A Whelton SP Wu CO Ambale-Venkatesh B Post WS. Association of lipoprotein(a) levels with myocardial fibrosis in the multi-ethnic study of atherosclerosis. J Am Coll Cardiol. (2023) 82(24):2280–91. 10.1016/j.jacc.2023.10.016 38057070 PMC11730445

[227] Weinreich M McDonough H Heverin M Domhnaill ÉM Yacovzada N Magen I. Optimised machine learning for time-to-event prediction in healthcare applied to timing of gastrostomy in ALS: a multi-centre, retrospective model development and validation study. EBioMedicine. (2025) 121:105962. 10.1016/j.ebiom.2025.105962 41075354 PMC12547709

[228] Yu J Lee D Kim N. Latent diffusion process with mechanistic guidance for designing functionally graded metamaterials with perfect connectivity. Adv Sci (Weinh)). (2026):e76914. 10.1002/advs.76914 42531599 PMC13423480

[229] Han H Sun C Wu X Yang H Zhao D. Self-Organizing stacked type-2 fuzzy neural network with rule generalization. IEEE Trans Neural Netw Learn Syst. (2025) 36(6):10128–42. 10.1109/tnnls.2025.3544495 40168224

[230] Kohda H Tanaka M Shichino S Arakawa S Komori T Ito A. Novel cell-to-cell communications between macrophages and fibroblasts regulate obesity-induced adipose tissue fibrosis. Diabetes. (2025) 74(7):1135–52. 10.2337/db24-0762 40063503 PMC12185969

[231] Qiang L Hoffman MT Ali LR Castillo JI Kageler L Temesgen A. Transforming growth factor-β blockade in pancreatic cancer enhances sensitivity to combination chemotherapy. Gastroenterology. (2023) 165(4):874–90.e10. 10.1053/j.gastro.2023.05.038 37263309 PMC10526623

[232] Quah Y Jung S Chan JY Ham O Jeong JS Kim S. Predictive biomarkers for embryotoxicity: a machine learning approach to mitigating multicollinearity in RNA-Seq. Arch Toxicol. (2024) 98(12):4093–105. 10.1007/s00204-024-03852-w 39242367

[233] Jhund PS. Use of NT-proBNP for the screening, diagnosis and risk-stratification of left ventricular dysfunction. Diabetes Obes Metab. (2025) 27 (Suppl 8):7–14. 10.1111/dom.16388 40205856 PMC12400483

[234] Shaukat A Levin TR Liang PS Weiss JM Smare C Boller E. Cost-effectiveness of novel noninvasive screening tests for colorectal neoplasia. Clin Gastroenterol Hepatol. (2026) 24(7):2007–18. 10.1016/j.cgh.2025.06.006 40562290

[235] Janes MJ Zhao L Bruton LG Schmidt AA Parker JM Wakefield MR. Rewiring immunity: CAR-T cells as a paradigm shift in hematologic oncology. Crit Rev Oncol Hematol. (2026) 222:105281. 10.1016/j.critrevonc.2026.105281 41845985

[236] Dutta A Chakraborty S Roy A Mittal A Basak T. Tissue fibrosis in cardiorenal syndrome: crosstalk between heart and kidneys. Nephrol Dial Transplant. (2025) 40(7):1273–83. 10.1093/ndt/gfaf009 39805714

[237] Chavarriaga J Langleben C Lobo J Nappi L Yousef GM Ozcan G. MicroRNA as a liquid biomarker to detect malignancy in small testicular masses. Eur Urol Oncol. (2025) 8(6):1575–82. 10.1016/j.euo.2025.08.004 40998685

[238] Li Y Wang J Ma D MicroRNA HJ. MicroRNA-lncRNA and MicroRNA-circular RNA axes, and exosomal MicroRNAs: driving exercise-induced cardioprotection in heart failure. Front Cell Dev Biol. (2026) 14:1767057. 10.3389/fcell.2026.1767057 41960190 PMC13057507

[239] Zhang Y Zhang W Wu Z Chen Y. Diversity of extracellular vesicle sources in atherosclerosis: role and therapeutic application. Angiogenesis. (2025) 28(3):34. 10.1007/s10456-025-09983-7 40522508

[240] Hiebert P Antoniazzi G Aronoff M Werner S Wennemers H. A lysyl oxidase-responsive collagen peptide illuminates collagen remodeling in wound healing. Matrix Biol. (2024) 128:11–20. 10.1016/j.matbio.2024.02.006 38382767

[241] Feng L Zhang F Cao J Xiong W. Role of cGAS-STING pathway in fibrotic disease. Pharmacol Res. (2025) 221:107971. 10.1016/j.phrs.2025.107971 41005587

[242] López B Ravassa S Moreno MU José GS Beaumont J González A. Diffuse myocardial fibrosis: mechanisms, diagnosis and therapeutic approaches. Nat Rev Cardiol. (2021) 18(7):479–98. 10.1038/s41569-020-00504-1 33568808

[243] Tayal U Verdonschot JAJ Hazebroek MR Howard J Gregson J Newsome S. Precision phenotyping of dilated cardiomyopathy using multidimensional data. J Am Coll Cardiol. (2022) 79(22):2219–32. 10.1016/j.jacc.2022.03.375 35654493 PMC9168440

[244] Murat-Onana ML Ramalingam SS Jänne PA Gray JE Ahn MJ John T. EGFR mutation testing across the osimertinib clinical program. Lung Cancer (Amsterdam). (2025) 204:108549. 10.1016/j.lungcan.2025.108549 40311309

[245] Guan X Wang J Feng Z Duan Z Zhang L Li Y. Alternating magnetic field-assisted freezing enhances the freeze-thaw stability of Pacific white shrimp (litopenaeus vannamei): evidence from physicochemical and metabolomic analyses. Food Chem. (2026) 507:148295. 10.1016/j.foodchem.2026.148295 41655493

[246] Pires IS Berthiaume F Palmer AF. Engineering therapeutics to detoxify hemoglobin, heme, and iron. Annu Rev Biomed Eng. (2023) 25:1–21. 10.1146/annurev-bioeng-081622-031203 37289555

[247] Zhang MY Yang ZH Qiu YC Gao YH Li SY Dang Y. LRG1, A novel serum biomarker for iMCD disease activity. Biomark Res. (2025) 13(1):56. 10.1186/s40364-025-00767-1 40197491 PMC11974158

[248] Nakamura M Huang GN. Why some hearts heal and others don’t: the phylogenetic landscape of cardiac regenerative capacity. Semin Cell Dev Biol. (2025) 170:103609. 10.1016/j.semcdb.2025.103609 40220599

[249] Zhu X Zhang J Zhou Z Liangpunsakul S Wang J Ge G. Huangjia ruangan granules with entecavir for fibrosis regression in chroinc hepatitis B: a multicenter, randomized, double-blind, phase II trial. Phytomedicine. (2025) 148:157434. 10.1016/j.phymed.2025.157434 41138575

[250] Begg GA Swoboda PP Karim R Oesterlein T Rhode K Holden AV. Imaging, biomarker and invasive assessment of diffuse left ventricular myocardial fibrosis in atrial fibrillation. J Cardiovasc Magn Reson. (2020) 22(1):13. 10.1186/s12968-020-0603-y 32036784 PMC7008543

[251] Huttin O Kobayashi M Ferreira JP Coiro S Bozec E Selton-Suty C. Circulating multimarker approach to identify patients with preclinical left ventricular remodelling and/or diastolic dysfunction. ESC heart Failure. (2021) 8(2):1700–5. 10.1002/ehf2.13203 33576580 PMC8006620

[252] Zhao T Wu X Zhao X Yao K Li X Ni J. Identification and validation of chemokine system-related genes in idiopathic pulmonary fibrosis. Front Immunol. (2023) 14:1159856. 10.3389/fimmu.2023.1159856 37122736 PMC10140527

[253] Liu XY Tang H Zhou QY Zeng YL Chen D Xu H. Advancing the precision management of inflammatory bowel disease in the era of omics approaches and new technology. World J Gastroenterol. (2023) 29(2):272–85. 10.3748/wjg.v29.i2.272 36687128 PMC9846940

[254] Sadee W Wang D Hartmann K Toland AE. Pharmacogenomics: driving personalized medicine. Pharmacol Rev. (2023) 75(4):789–814. 10.1124/pharmrev.122.000810 36927888 PMC10289244

[255] Peng HT Siddiqui MM Rhind SG Zhang J da luz LT Beckett A. Artificial intelligence and machine learning for hemorrhagic trauma care. Mil Med Res. (2023) 10(1):6. 10.1186/s40779-023-00444-0 36793066 PMC9933281

[256] Liu J Yang L Liu D Wu Q Yu Y Huang X. The role of multi-omics in biomarker discovery, diagnosis, prognosis, and therapeutic monitoring of tissue repair and regeneration processes. J Orthop Translat. (2025) 54:131–51. 10.1016/j.jot.2025.07.006 40822515 PMC12356027

[257] Wang C Lyu J Wang S Qin C Guo K Zhang X. CMRxrecon: a publicly available k-space dataset and benchmark to advance deep learning for cardiac MRI. Sci Data. (2024) 11(1):687. 10.1038/s41597-024-03525-4 38918497 PMC11199635

[258] Bellomo G Vermunt L In ‘t Veld S Doecke JD Hok AHYS Veverová K. Plasma proteome profiling identified biomarkers for the differential diagnosis and molecular staging of neurodegenerative dementias. Nat Aging. (2026) 6:1717–32. 10.1038/s43587-026-01162-7 42477094 PMC13472907

[259] De Zawadzki A Leeming DJ Sanyal AJ Anstee QM Schattenberg JM Friedman SL. Hot and cold fibrosis: the role of serum biomarkers to assess immune mechanisms and ECM-cell interactions in human fibrosis. J Hepatol. (2025) 83(1):239–57. 10.1016/j.jhep.2025.02.039 40056933

[260] Tang X Leng M Tang W Cai Z Yang L Wang L. The roles of exosome-derived microRNAs in cardiac fibrosis. Molecules (Basel). (2024) 29(6):1199. 10.3390/molecules29061199 PMC10974027 38542836

[261] Miller CS Ding X Nagarajan R Dawson DR 3rd Ebersole JL. Biomarker panel discriminates diabetics with and without periodontitis pre- and post-therapy. J Periodontal Res. (2023) 58(3):493–502. 10.1111/jre.13127 37042710

[262] Lengvenyte A Blaquiere M Dubois J Bonnin M Bourret J Hamzeh-Cognasse H. Integrating cellular and soluble immune signatures of major depression with and without recent suicide attempts. Transl Psychiatry. (2025) 15(1):377. 10.1038/s41398-025-03601-2 41053103 PMC12501231

[263] Nissa MU Pinto N Ghosh B Banerjee A Singh U Goswami M. Proteomic insights into extracellular matrix dynamics in the intestine of labeo rohita during Aeromonas hydrophila infection. mSystems. (2024) 9(10):e0024724. 10.1128/msystems.00247-24 39292008 PMC11495024

[264] Huang H Liu S Yao J Bai X Jin Z Xue B. Endothelial MerTK impairment promotes cardiac dysfunction in the condition of high fat diet. Redox Biol. (2026) 93:104184. 10.1016/j.redox.2026.104184 42030645 PMC13125900

